# Structure-Function Relationship of the Disintegrin Family: Sequence Signature and Integrin Interaction

**DOI:** 10.3389/fmolb.2021.783301

**Published:** 2021-12-03

**Authors:** Ariana A. Vasconcelos, Jorge C. Estrada, Victor David, Luciana S. Wermelinger, Fabio C. L. Almeida, Russolina B. Zingali

**Affiliations:** ^1^ Instituto de Bioquímica Médica (IBqM) Leopoldo de Meis, Centro Nacional de Ressonância Magnética Nuclear, Universidade Federal do Rio de Janeiro, Rio de Janeiro, Brazil; ^2^ Centro Nacional de Ressonância Magnética Nuclear (CNRMN), Centro Nacional de Biologia Estrutural e Bioimagem (CENABIO), Universidade Federal do Rio de Janeiro, Rio de Janeiro, Brazil; ^3^ Laboratório de Hemostase e Venenos, Instituto de Bioquímica Médica (IBqM) Leopoldo de Meis, Universidade Federal do Rio de Janeiro, Rio de Janeiro, Brazil; ^4^ Faculdade de Farmácia, Universidade Federal do Rio de Janeiro, Rio de Janeiro, Brazil

**Keywords:** snake venom disintegrin, structure, integrin, NMR, crystallography

## Abstract

Disintegrins are small cysteine-rich proteins found in a variety of snake venom. These proteins selectively modulate integrin function, heterodimeric receptors involved in cell-cell and cell-matrix interaction that are widely studied as therapeutic targets. Snake venom disintegrins emerged from the snake venom metalloproteinase and are classified according to the sequence size and number of disulfide bonds. Evolutive structure and function diversification of disintegrin family involves a stepwise decrease in the polypeptide chain, loss of cysteine residues, and selectivity. Since the structure elucidation of echistatin, the description of the structural properties of disintegrins has allowed the investigation of the mechanisms involved in integrin-cell-extracellular matrix interaction. This review provides an analysis of the structures of all family groups enabling the description of an expanded classification of the disintegrin family in seven groups. Each group presents a particular disulfide pattern and sequence signatures, facilitating the identification of new disintegrins. The classification was based on the disintegrin-like domain of the human metalloproteinase (ADAM-10). We also present the sequence and structural signatures important for disintegrin-integrin interaction, unveiling the relationship between the structure and function of these proteins.

## Introduction

Integrin antagonists comprise molecules that can bind and interfere with the activity of the cellular receptors integrins. Some proteins or peptides are found in snake venoms such as the C-type lectins EMS6 (Marcinkiewicz et al., 2000) and Vixapatin (Momic et al., 2012) or disintegrins (Calvete, 2005a), or found in other animals venoms such as arthropods (Khamessi et al., 2018). The snake venom disintegrin family comprises a group of cysteine-rich proteins (40–100 amino acids) found in the venom from snakes from Elapidae, Viperidae, Atractaspididae, and Colubridae families (Kini and Evans, 1992; Calvete et al., 2005b; Arruda Macedo et al., 2015). These proteins are released in the venom as a result of the proteolytic process of the so-called PII snake venom metalloproteinases (SVMP) (Kini and Evans, 1992; Calvete et al., 2005b). It is worth emphasising that venoms from other venomous animals also contain metalloproteinases similar to that from snake venoms (Xia et al., 2013; Borges et al., 2016). Whether these molecules are able to generate disintegrins is yet to be elucidated. Snake venoms disintegrins were firstly described by the capacity to inhibit the platelet fibrinogen receptor integrin αIIbβ3 (Huang et al., 1987). Disintegrins are capable of modulating the function of a broad range of integrins (Gould et al., 1990; McLane et al., 2004), a family of heterodimeric receptors that play a fundamental role in mediating physiological and pathological processes, such as hemostasis and cancer (Phillips et al., 1980; Hynes, 1987). Many aspects of the disintegrin protein family were reviewed recently: as tools for antitumor activity (Schönthal et al., 2020), for antithrombotic agents (Kuo et al., 2019), recombinant and chimeric proteins on research (Huang et al., 2016; David et al., 2018; Uzair et al., 2018; Cesar et al., 2019; Lazarovici et al., 2019). This review focuses on structural studies of snake venom disintegrins and the features of integrin-disintegrin interaction.

## Structural and Evolutive Bases of Disintegrin Molecules

### Evolutive Diversification of Metalloproteinases Into Disintegrins

The emergence of disintegrins results from the divergence of snake venom Zn^2+^-Metalloproteinases (SVMPs). These proteins are present in the venom of the majority of venomous snakes and are capable of degrading the extracellular matrix and/or proteins belonging to the hemostasis system (de Lima et al., 2009; Fox and Serrano, 2009; Mackessy, 2009). SVMPs share a common ancestor with matrix-degrading metalloproteinases, the A Disintegrin And Metalloproteinase (ADAM) family (Moura-da-Silva et al., 1996). SVMPs are classified according to their domain organization: the PIII class contains the metalloproteinase domain followed by the C-terminal disintegrin-like and cysteine-rich domain; the PII class, contains the disintegrin domain at the C-terminal of the metalloproteinase domain and the PI class contains only the metalloproteinase domain (Fox and Serrano, 2009). The subfamily of ADAM containing the thrombospondin type-1 motif (ADAMTS) are also related to SVMPs, but the crystallographic structures showed that the disintegrin-like domain of ADAMTS adopts a different fold and are not structurally homologous to disintegrins (Takeda et al., 2012). For this reason, the analysis presented in this review will consider only the ADAM family.

The evolution of disintegrin family was extensively studied by many authors (for review see: Juárez et al., 2008; Calvete, 2013; Fox and Serrano, 2009). The accepted hypothesis is that SVMPs have evolved relatively late from a common ancestor by speciation and positive Darwinian selection. The evolution started by gene duplication of the ancestral PIII disintegrin-like domain followed by neofunctionalization in the snake venoms generating the disintengrin domains of PII (Fox and Serrano, 2009). The evolution from disintegrin-like (PIII) to disintegrin domain (PII) ocurred through the successive loss of disulfide bonds and reduction in size to the different snake venom disintegrin subfamilies (long, medium-sized, dimeric, and short (Kini and Evans, 1992; Calvete, 2005a). The emergence of PII SVMP precursors follows another key event that includes deletion of the C-terminal cysteine-rich domain, due to a mutation causing the appearance of a stop codon, and the removal disulfide bonds (Calvete et al., 2003; Juárez et al., 2006a). PII SVMP undergoes limited proteolysis, releasing disintegrins domains (short, medium, and long) into the venom (Kini and Evans, 1992; Jia et al., 1996). Most proteins from dimeric or short groups are synthesized from short-coding mRNAs, lacking the metalloproteinase domain (Juárez et al., 2006b; Okuda et al., 2002; Bazaa et al., 2007). In fact, analysis of the pre-sequence of the cDNA disintegrin jerdostatin suggests that this disintegrin originates from a short-coding gene, instead of a proteolytic process such as the majority of disintegrin from PII SVMP (Sanz et al., 2005). Moreover, Bazaa et al., 2007 suggested that the shortening of the gene is due to the loss of introns and coding regions that contribute to the formation of the short-coding disintegrins. In summary, disintegrins originate from a multigene family from metalloproteinases that undergoes an accelerated evolutionary pathway resulting in great diversification (Juárez et al., 2008).

### Structural Diversification of Disintegrins

**FIGURE 1:**
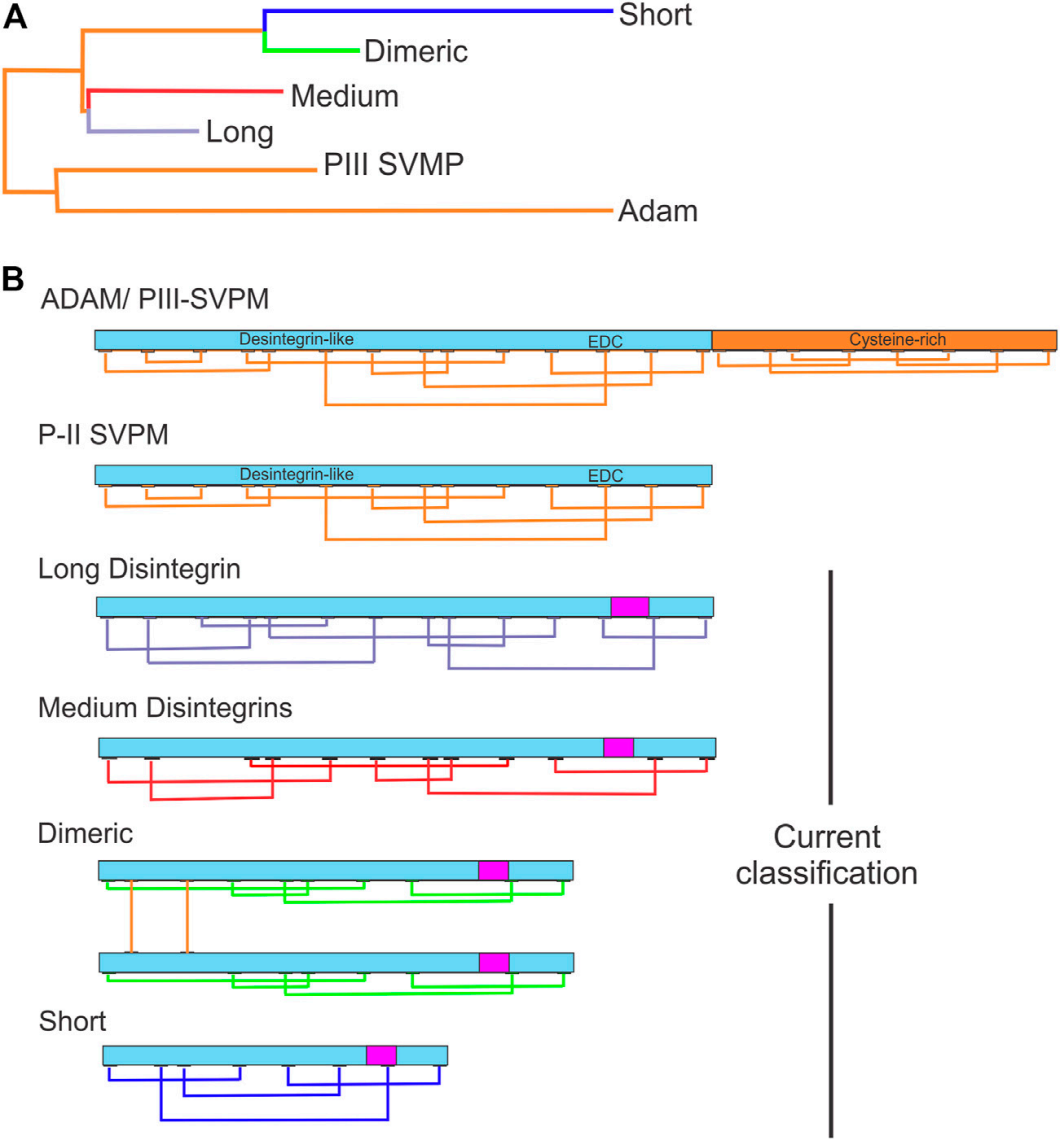
Evolutionary relationships between disintegrins and current classification. **(A)** Dendrogram shows the evolutionary relationships between the different disintegrin subfamilies. The dendrogram also includes the disintegrin-like domain of ADAMs and PIII-SVMPs from which the different snake venom disintegrin subfamilies (long, medium, dimeric and short) evolved through the successive loss of disulfide bonds and size reduction. **(B)** Current classification of disintegrins: long (∼84 amino acids and 7 disulfide bonds) (connections between cysteines in lavender), medium (∼70 amino acids and 6 disulfide bonds) (connections between cysteines in red), dimeric (∼67 amino acids and 4 intrachain disulfide bonds for each subunit and 2 interchain) (connections in green), and short (41–51 amino acids and 4 disulfide bonds) (connections in blue). The integrin-binding RGD, KGD, MGD, MDL, KTS, and RTS tripeptide motifs localization is indicated by the magenta rectangle. (Adapted from Juaréz et al., 2008).

Disintegrins have been classified according to the number of residues and disulfide bridges, into four subfamilies: smaller ones are the short disintegrins composed of 49–51 residues and four disulfide bridges; the medium size disintegrins with about 70 residues and six bridges; the largest consist in the long disintegrin with about 84 residues cross-linked by seven disulfide bridges; and homo- and heterodimers disintegrins (McLane et al., 1998; Calvete et al., 2003). Dimeric disintegrins contain subunits about 67 residues cross-linked by 2 interchain cysteine linkages and 4 intra-chain disulfide bonds (Calvete et al., 2000; Bilgrami et al., 2005; Figure 1). The evolution pathway of disintegrin structure diversification involved the reduction of the polypeptide chain and selective loss of pairs of cysteine residues that form disulfide bonds (Calvete et al., 2003; Calvete, 2005a, 2010; Bazaa et al., 2007). Phylogenetic analysis suggests that PII-dimeric and short disintegrins represent the more recent diverging lineages of disintegrins (Figure 1A) (Juárez et al., 2008).

Additionally, disintegrins present much more complex structural diversity. To illustrate, disintegrins jarastatin (*Bothrops jararaca*)^1^, triflavin (*Trimeresurus flavoviridis*), and obtustatin exhibit different disulfide bridge patterns and structural features. Jarastatin and triflavin are disintegrins that contain six disulfide bonds with the same cysteine pattern, however, these disintegrins have different cystine connectives (Huang T. F. et al., 1991; Coelho et al., 1999; Cidade et al., 2006). Likewise, triflavin has an elongated and rigid structure composed of turns and antiparallel β-strands, and obtustatin has a compact globular structure composed of turns and without regular secondary structure (Huang T. F. et al., 1991; Coelho et al., 1999; Fujii et al., 2003; McLane et al., 2004; Cidade et al., 2006; Wermelinger et al., 2009). These cysteine pairing characteristics motivated us to perform a re-analysis of the classification of disintegrins based on the 3D structure to seek a better structural/function comprehension of disintegrins (see point section 4.1).

Moreover, despite the majority of disintegrins following canonical structure features, some of them follow a different pathway (Calvete et al., 2003). The disintegrin graminelysin belongs to medium-size disintegrins but has some features of PIII derived disintegrins, such as the Cys13-Cys16 disulfide bond, which represent an intermediate step in the evolution pathway of medium-sized disintegrins (Wu et al., 2001). Bilitoxin-1 is a long disintegrin containing an additional cysteine residue involved in a disulfide bond homodimer (Nikai et al., 2000). Another example includes the identification of different PII SVMP transcripts found in the venom of *Bothrops neuwiedi*, suggesting recombination of PII SVMP with the catalytic domain of PI or PIII SVMP (Moura-da-Silva et al., 2011). This fact is well described below, where the groups are presented based on structural and cysteine pairing features. We also show a cladogram that illustrates the evolutionary path of the disintegrins.

## Disintegrin Structure: NMR and Crystallization Studies

**FIGURE 2:**
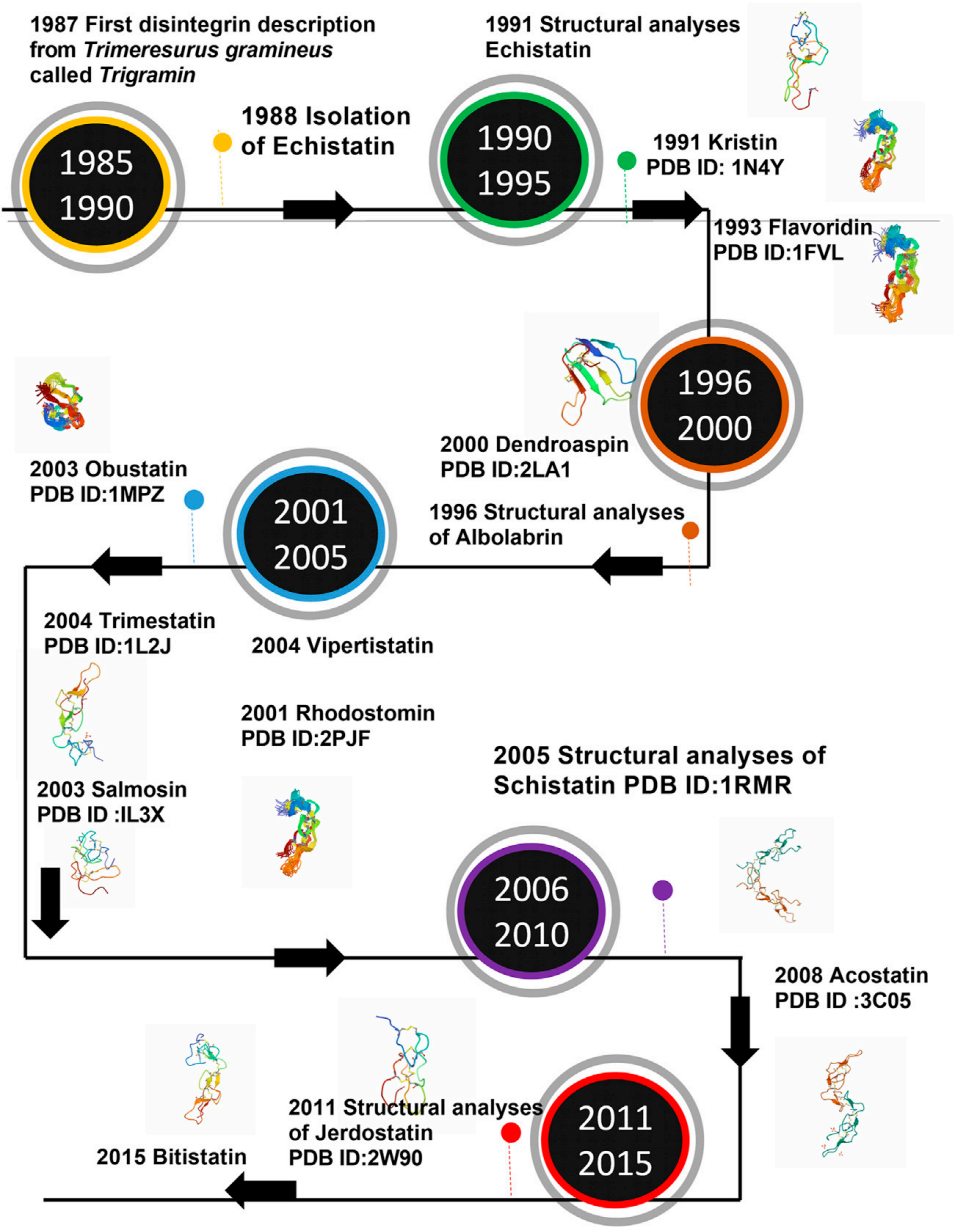
Timeline of the structure determination of Disintegrins. In a time-lapse of 5 years from the first disintegrin discovery.

The number of disintegrins purified from the venom or identified by proteome or transcriptome analysis grows each year. The NMR studies expanded the panorama of the structure of disintegrins. Nevertheless, the structure of only 16 disintegrins has been solved by NMR or crystallography until now. Figure 2 presents the timeline of experimental information on disintegrin structure.

### An Expanded Classification of Disintegrins Based on the Structural Features, Disulfide Pattern, and Comparison With Ancestral Disintegrin Fold

In this section, we propose an expanded classification based on the structural features of disintegrins and use the disintegrin-like domain of a PIII ADAM as a reference for folding and disulfide pattern. This reference is suitable since it is well established by several in-death phylogenetic studies (Juárez et al., 2008; Fox and Serrano, 2009; Calvete, 2013) that the PIII disintegrin-like domain is ancestral of all disintegrins. As previously mentioned in this work, disintegrins have a vast structural diversity. Within the disintegrin family there is a wide variety of disulfide bridge patterns and protein sizes and, at the same time, conserved structural features (Huang T. F. et al., 1991; Coelho et al., 1999; Cidade et al., 2006; Cheng et al., 2012). For instance, jarastatin and triflavin disintegrins contain six disulfide bonds with the same cysteine pattern while the disintegrin domain of ADAM metalloproteinases present the same size, structural homology, and different disulfide pattern (Huang T.-F. et al., 1991; Coelho et al., 1999; Cidade et al., 2006).

Pairing cysteine residues in disintegrins is already known to play an important role in exposing the RGD binding motif that mediates inhibition of platelet aggregation, neutrophils, or endothelial cell function (Blodel and White, 1992; Takagi et al., 2002; Calvete et al., 2003; Calvete, 2005a). In addition, the modulatory activity of the disintegrins depends on the proper pairing of the cysteine residues, contributing to the conformation of the disintegrin structure (Niewiarowski et al., 1994). The conservative aspect of cysteine residues and the disulfide bond pattern between the disintegrins subfamily contribute to the hypothesis of strong selection for maintaining the active conformation of these proteins (Juárez et al., 2008). Therefore, we decided to investigate in more detail the patterns of protein disulfide bridges within the structure of some members of the disintegrin family, which show disintegrin fold either as an isolated protein or as a domain of a larger protein.

**FIGURE 3:**
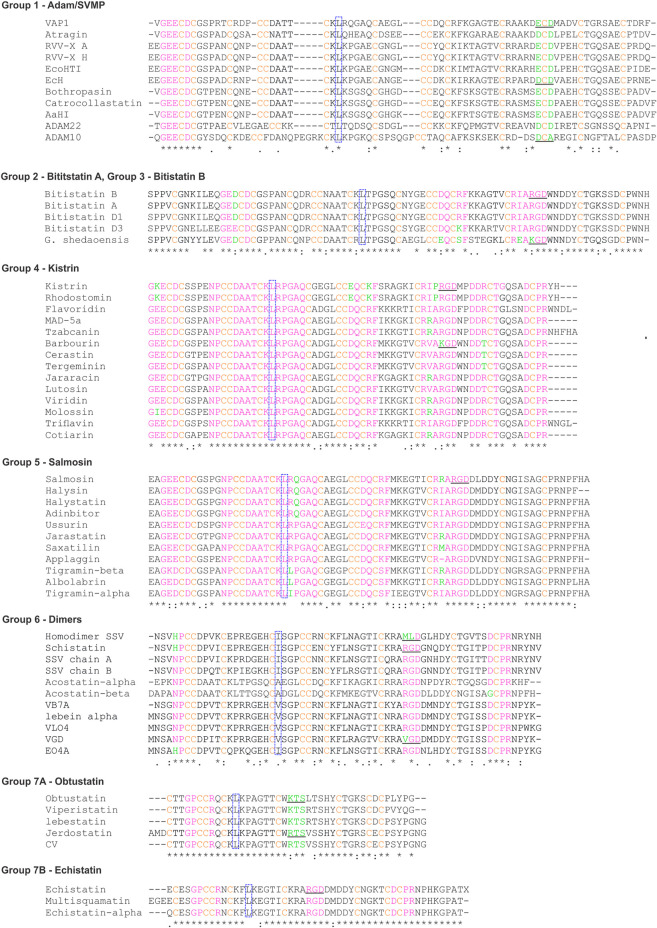
Sequence alignments of representative disintegrins. The sequences were obtained through research in the Blast software (Altschul et al., 1990; Gish and States, 1993). The pink color highlights the sequence signatures, green the residues that are not conserved within the signatures, orange the cysteines. Dotted blue squares show the conserved L/I that compose the hydrophobic core. RGD/KGD or related three-peptides are underlined. The codes representing the sequences were obtained from PDB and UniProt. Abbreviated: VAP-1 (Vascular apoptosis-inducing protein 1); RRV-X (Russell’s viper venom factor X activator); EcoHIT (Viper PIII hemorrhagic SVMP *Echis coloratus*); EcH (Metalloproteinase *Echis pyramidum leakeyi*); *Gloydius shedaoensis* (*G. shedaoensis*); SSV (saw-scaled Viper). The multiple sequence alignment was performed using the ClustalW2 server (https://www.genome.jp/tools-bin/clustalw).

To select these proteins, we used the PFAM family (PF0020) classification, which describes all the subfamilies and their correspondent structures, contains annotations and multiple sequence alignments. We analyzed the sequence and structure alignment and proposed a classification based on the pattern of disulfide bonds using the ancestral subfamily ADAM as a reference. We assigned letters from A to N for each possible position occupied by these cysteines. The analysis pointed out seven possible disulfide patterns, named group 1, for proteins of ADAM subfamily; group 2, for disintegrins similar to bitistatin A; group 3, bitistatin B; group 4, for kistrin; group 5 for salmosin; group 6, for dimers; group 7, for obtustatin. Figure 3 shows the sequence alignment of each group proposed in this classification.

**FIGURE 4:**
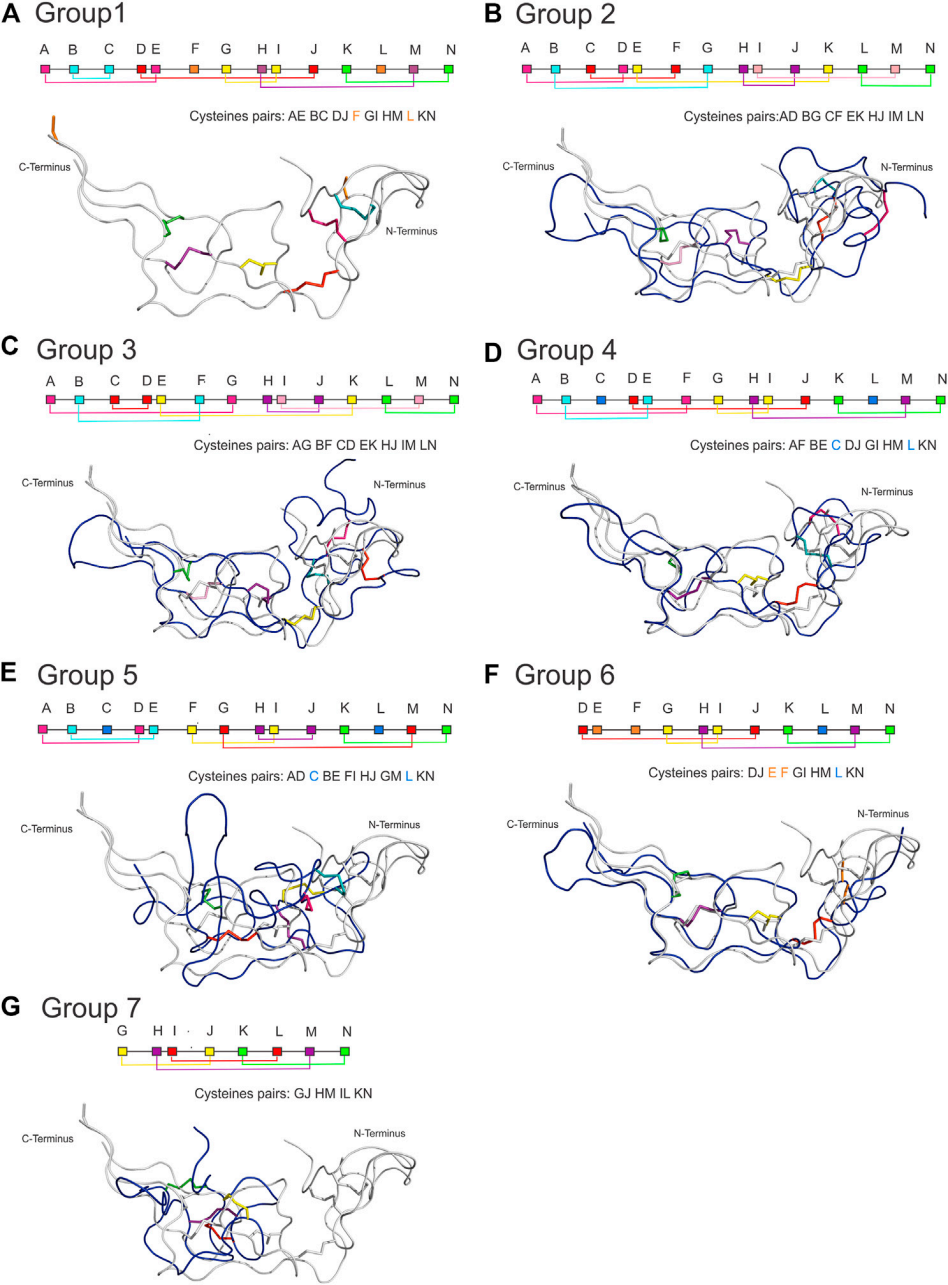
Disulfide bonding pattern for each group and the structural superposition of a component of each group with ADAM10. Each group has a characteristic pattern that allowed us to be divided into seven groups, where **(A)** Group 1 of the ADAM/SVPM; **(B)** Group 2 of Bitistatin A; **(C)** Group 3 of Bitistantin B; **(D)** Kistrin Group 4; **(E)** Group 5 of Salmosin; **(F)** Group 6 of the dimers; **(G)** Group 7 of the Obtustatin/Echistatin (here represented by Obtustatin). In the connectivity scheme, each cysteine is represented by a letter that follows the order from A to N with different colors, where cysteines involved in interchain connections or connections between domains can occur (orange square), and still, cysteines replaced by another amino acid residue (light blue square). (Superposition color). Note that the structures of all groups can be superposed, except for salmosin, which shows a different conformation at the N-terminus.

The classification of the disulfide bond patterns followed the tertiary structure alignments with the reference protein ADAM10 Extracellular Domain (ADAM10, PDB id 6BE6). Only the alignments of primary structure did not provide sufficient information to classify the pattern within the disintegrin family, whereas the comparison with ADAM10 provided the exact location and classification for each cysteine. Figure 4 shows the disulfide pattern for each group and the superposition of one component of each group with ADAM10. It illustrates the rationale of the classification proposed here.

For group 1 (Figure 4A), we analyzed 11 representatives, which have a disintegrin domain with up to 14 cysteines. They are organized into 6 disulfide bonds and two cysteines that may form disulfide bonds with cysteines in the other domain of the protein (Jia et al., 1996). The letter-based pattern is defined as AE BC DJ F GI HM L kN, where F and L, which appear here unmatched, are cysteines involved in connections with the other domains. This group is formed by mammalian (ADAM) and snake venom metalloproteinases (SVMP), both containing the disintegrin domain with the same fold.

Group 2 and 3 represent two disulfide patterns (alternate folding) for the same sequence. They were first described for the disintegrin bitistatin, group 2 for bitistatin A (Figure 4B), and 3 for bitistatin B (Figure 4C). The disulfide pattern for group 2 is AD BG CF EK HJ IM LN and AG BF CD EK HJ IM LN for group 3 differing only at disulfide bonds at the N-terminal region.

Group 4 (Figure 4D) is the group of kistrin, which is also presented as only 1 disintegrin monomeric domain. It consists of 15 proteins that have 12 cysteines, all involved in disulfide bonds. The pattern is AF BE DJ GI HM KN with the cysteines of positions C and L were evolutionarily replaced by other amino acids. We noted that the disulfide pattern is the same as that found for the ancestral subfamily ADAM, differing only at the N-terminal region. Despite this, the detailed analysis of the sequence alignment (Figure 3) of the studied groups showed that the ADAM group (group 1) and the Kistrin group (group 4), have many conserved amino acid sequences in the N-terminal portion.

Group 5 (Figure 4E) are also monomeric proteins with 1 disintegrin domain. It consists of 11 proteins, but the only member with a high-resolution structure available is the salmosin. This disintegrin has a different disulfide pattern, represented by AD BE FI HJ GM KN. Interestingly, the cysteines that occupied the C and L positions in the ADAM ancestor were replaced by other amino acids throughout the evolutionary process and for this reason, they are not included in this pairing. Therefore, members of this group include 12 cysteines involved in 6 disulfide bonds. Salmosin is the only available disintegrin structure solved so far that cannot be completely superposed, differing at the N-terminal region, with the reference group (group 1) and other disintegrins.

Group 6 (Figure 4F) are the dimeric disintegrins. They include 4 representatives with resolved structures. The dimeric disintegrins are small and present the N-terminal region quite conserved among themselves, but distinct when compared to the other disintegrins. They have 10 cysteines, of which 8 are involved in intra-chain disulfide bonds and two are involved in inter-chain cystine linkages (cysteines E and F). Also, they present evolutionary replacement at the L position for other amino acids. The pattern observed for this group is DJ EF GI HM KN, containing the sequence signature NPCC at the N-terminal (Figure 3 and Table 1).

**TABLE 1:** Signatures of the sequences of the study groups based on the classification of seven groups.

Sequence	G1	G2	G3	G4	G5	G6	G7A	G7B
GxECDC	✔	✔	✔	✔	✔	—	—	—
CCDAATCKLxxGAQC	—	—	—	✔	✔	—	—	—
GPCCR	—	—	—	—	—	—	✔	✔
CRxARGD	—	✔	✔	✔	✔	—	—	—
CCxQCxF	—	✔	✔	✔	—	—	—	—
DDxCxG	—	—	—	✔	✔	—	—	—
DCPR	—	—	—	✔	—	✔	—	✔
NxCC	—	—	—	✔	✔	✔	—	—

x variation of amino acid residue.

Group 7 (Figure 4G) comprises the small disintegrins, which have members that have, in addition to the RGD motif, KTS, and RTS sequences, with a GJ HM IL KN binding pattern, these disintegrins have 8 cysteines that form 4 disulfide bonds. In addition, group 7 displays few sequence signatures in common with other groups (Table 1).

The new classification of the disintegrin family provided the description of the sequence signatures that will enable localization of an unknown sequence within a group and to be able to predict properties such as structural features and functional capabilities. We looked for sequence signatures within each group that may help to classify and model the structure of an unknown sequence. The signature GxECDC sequence is common for the groups G1, G2, G3, G4, and G5, absent in groups G6 and G7 (short and dimeric disintegrins, Table 1). Here, we emphasize that group 7 was subdivided into 7A and 7B.

The sequences CRxARGD and CCxQCxF are common to groups G2, G3, G4, and G5, which relate to medium and long proteins. The CRxARGD signature is shared with the salmosin group, a more distinct group due to its more differentiated disulfide bond pattern.

We also observed signatures present in some related groups, such as CCDAATCKLxxGAQC and DDxCxG, which were common to groups G4 and G5. Both groups harbor medium-sized disintegrins, but with different folds. We also observed that short disintegrins exhibit the highest variability in their integrin recognition motifs, including, in addition to RGD, the KTS and RTS motifs. This variability in the motifs for integrin recognition was also observed for G6, a group of dimeric disintegrins, which present in addition to RGD, the motifs MDL and VGD and share the DCPR signature at the c-terminal portion with the G4 groups of Kistrin and and some members of G7B Echistatin.

**FIGURE 5:**
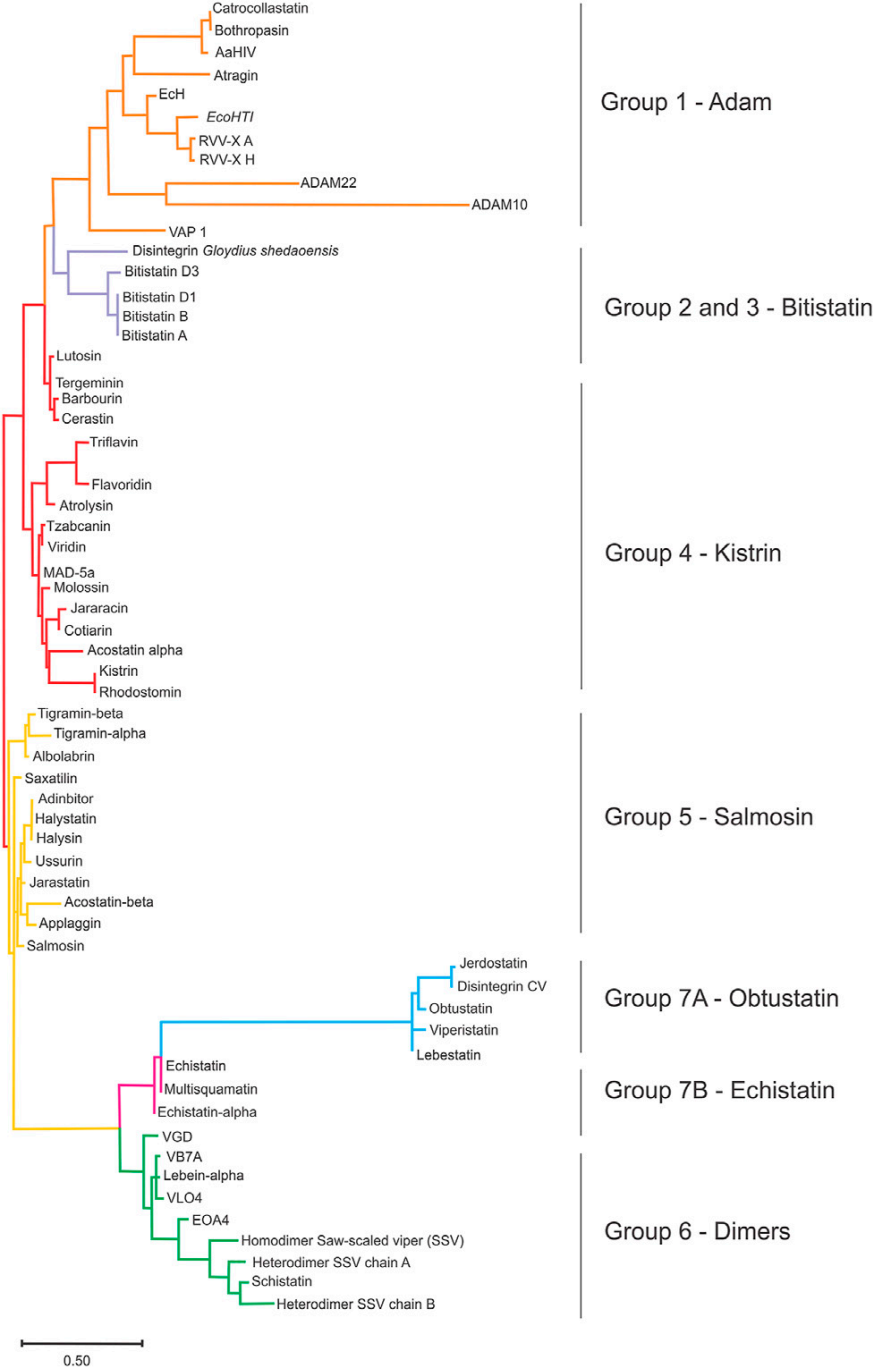
The sequence clustering of disintegrin. Simplified neighbor-joining method, revealing the main sequence clusters (clades) of the disintegrins using the ClustalW2 server (https://www.genome.jp/tools-bin/clustalw) and MEGA software (Molecular Evolutionary Genetics Analysis) (Kumar et al., 2018) as a tool for sequence alignment, and clustering. This analysis report is useful for sequence clustering in clades, but not for the temporal evolution analysis. The goal of presenting the cladogram is to support the classification. The ancestor of an ADAM gene (orange), and the emergence of the disintegrin family through the successive loss of disulfide bonds. The cysteine residues that are lost along the evolutionary pathway result in divisions in disintegrins long (lavender) that comprises groups 2 and 3; medium disintegrins (red), group 4; medium disintegrins with a non-canonical disulfide pattern, which has Salmosin as a representative of group 5 (yellow); dimeric disintegrins, gathered here in group 6; and short disintegrins divided into groups 7A, Obtustatin group (blue) and group 7B, Echistatin group (pink).

We present a simple cladogram analysis based solely on the alignment of the amino acid sequences presented in Figure 3 using Mega (Kumar et al., 2018) (Figure 5), which showed a good correlation with the classification of disintegrins based on their structural properties (Figure 4) and corroborating previous evolutionary and structural studies (Calvete et al., 2003; Calvete, 2005a). We want to make it clear that the cladogram showed in Figure 5 reports well the clade of each group, but not the temporal evolution line. For an in-depth phylogenetic analysis, see Juárez et al., 2008, Calvete, 2013 and Fox and Serrano, 2009. The goal of presenting the cladogram is to support the classification. This analysis, together with data from the literature, showed that the evolutionary process that resulted in structural and functional diversification within the family of disintegrins, involved a reduction in the number of cysteine residues and successive losses of disulfide bonds (Juárez et al., 2008; Calvete, 2010; Carbajo et al., 2015). The different disulfide bond patterns (Figure 4) highlighted the idea that the disintegrins represent an example of the divergent evolution of a conserved structural motif (Carbajo et al., 2015).

### Disintegrin Structural Properties

Since the purification of Echistatin, isolated for the first time in 1988 from the venom of *Echis carinatus* snake, some disintegrins’ structures were solved leading to a better understanding of the structure-activity relationship. As mentioned before, the disintegrins vary in size and fold, but they display many conserved structural features. Table 2 summarizes the information about all disintegrins that had their structure solved so far. They are stabilized by multiple disulfide bonds (from 4 to 7), they lack a canonical hydrophobic core and well-defined secondary structures.

**TABLE 2:** Summary of the sequence, ligands, structural information and references of the disintegrins structures reported in PDB database.

Disintegrin name	Length (aa)	Number of cysteines	Ligand	Specie	Loop	Method	RMSD resolution (Å)	PDB ID	Group	References
Bitistatin	89	14	αvβ3	*Bitis arietans*	RGD	NMR	0.50	2MOP/2MP5	2–3	Carbajo et al. (2015)
Kistrin	68	12	αvβ3	*Agkistrodon rhodostoma*	RGD	NMR	0.47	1N4Y	4	Cooke et al. (1992)
Rhodostomin	68	12	αvβ3/αIIbβ3	*Calloselasma rhodostoma*	RGD	NMR	0.61	2PJF	4	Guo et al. (2001)
Rhodostomin R46E mutant	68	12	αIIbβ3	*Calloselasma rhodostoma*	RGD	CRYSTAL	NA	4R5U	4	Huang et al. (2015a)
Rhodostomin D51E mutant	68	12	αvβ3	*Calloselasma rhodostoma*	RGD	NMR	0.59	2PJG/2PJF	4	Chen et al. (2009)
Rhodostomin P48A mutant	68	12	αvβ1	*Calloselasma rhodostoma*	RGD	NMR	0.57	2PJI	4	Chuang et al. (2007)
Rhodostomin G50L mutant	68	12	αvβ3	*Calloselasma rhodostoma*	RGD	NMR	0.45	2LJV	4	Chuang et al. (2012)
RhodostominARGDP mutant	68	12	αIIbβ3/αvβ3	*Calloselasma rhodostoma*	RGD	CRYSTAL	NA	4M4C	4	Chang et al. (2014)
RhodostominKKKRT mutant	68	12	αIIbβ3/αvβ3	*Calloselasma rhodostoma*	RGD	CRYSTAL	NA	4R5R^b^	4	Huang et al. (2015b)
Rhodostomin ARLDDL mutant	72	12	αvβ3	*Calloselasma rhodostoma*	RGD	CRYSTAL	NA	3UCI	4	Shiu et al. (2012)
Disintegrin with the AKGDWN motif	68	12	αIIbβ3	*Calloselasma rhodostoma*	RGD	NMR	0.75	1Q7I	4	Chuang et al. (2004)
Rhodostomin mutante Protein RGD Motif C-terminal mutation	79	12	αIIbβ3	*Calloselasma rhodostoma*	RGD	NMR,	0.54	2M7F	4	Chang et al. (2014)
Rhodostomin mutante Protein RGD Motif C-terminal mutation	79	12	αIIbβ3	*Calloselasma rhodostoma*	RGD	NMR	0.51	2M7H	4	Chuang et al. (2013)
Rhodostomin mutante Protein RGD Motif C-terminal mutation	76	12	αIIbβ3	*Calloselasma rhodostoma*	RGD	NMR	0.60	2M75	4	Chuang et al. (2013)
Flavoridin	70	12	αIIbβ3	*Trimeresurus flavoviris*	RGD	NMR	0.85	1FVL	4	Senn and Werner, (1993)
Trimestatin	70	12	αvβ3	*Trimeresurus flavoviris*	RGD	CRYSTAL	NA	1J2L	4	Fujii et al. (2003)
Acostatin	64	10	αIIbβ3	*Agkistrodon contortrix contortrix*	RGD	CRYSTAL	NA	3C05	4–5	Moiseeva et al. (2008)
Albolabrin	84	12	αIIbβ3	*Trimeresurus albolabris*	RGD	NMR	NA	NA	5	Smith et al. (1996)
Salmosin	70	12	αIIbβ3	*Agkistrondon halys*	RGD	NMR	0.49	1L3X	5	Shin et al. (2003)
Schistatin	64	10	αIIbβ3/αvβ3	*Echis carinatus*	RGD	CRYSTAL	NA	1RMR	6	Bilgrami et al. (2004)
Heterodimer from Saw-scaled viper	64	10	αIIbβ3/αvβ3	*Echis carinatus*	RGD	CRYSTAL	NA	1TEJ	6	Bilgrami et al. (2005)
Saw-scaled viper	64	10	α4β1/α4β7	*Echis carinatus*	MLD	CRYSTAL	NA	1Z1X	6	Hassan et al. (2005)
Jerdostatin	46	8	α1β1	*Protobothrops jerdonii*	RTS	NMR	0.64	2W9O	7A	Carbajo et al. (2011)
Viperistatin	44	8	α1β1	*Vipera palestinae*	KTS	NMR	NA	NA	7A	Kisiel et al. (2004)
Obtustatin	41	8	α1β1	*Vipera lebetina obtusa*	KTS	NMR	0.46	1MPZ	7A	Moreno-Murciano, (2003)
Echistatin	49	8	αIIbβ3	*Echis carinatus*	RGD	NMR	0.52	2ECH/IRO3/6LSQ	7B	Chen et al. (1991)

The disintegrins structure consists of a series of loops tightly held together by disulfide bonds with almost no regular secondary structure. All hydrophobic residues are at least partially exposed to the protein surface, except for one leucine/isoleucine residue that is buried in a protein core. This I/L residue is present in all groups and should be important for protein stabilization. More studies are necessary to understand the role of the hydrophobic residues in the overall folding. The exposure of hydrophobic residues and folding without a canonical hydrophobic core is not exclusive for disintegrins and it is also present in defensins (Machado et al., 2018; Pinheiro-Aguiar et al., 2020). It was recently proposed that these proteins are stabilized by hydrophobic surface clusters acting as an independent folding unit. In the absence of a canonical hydrophobic core, the surface clusters promote the folding by the interaction of exposed hydrophobic residues with the adjacent side chains regulated by solvation forces (Almeida et al., 2021).

There are exceptions for the disintegrin general structure. One exception is bitistatin, which is mainly found in two different forms, as bitistatin A, and B. Both have an identical primary structure, their molecular architecture can be defined, generally, as a fold with an elongated shape, including a “ladder” of seven disulfide bonds, representing the dominant organizational characteristic of polypeptide folds. Some differences are found in the intra-domain interactions between the two bitistatins. In bitistatin A, some hydrophobic interactions are present between Val4-Pro21 and between Ile9 and the fragment Glu11-Cys18. Also, some inter-domain interactions are found with the aromatic ring of Tyr44 and the side chain of the sequence Ile9-Glu14, and between Leu36 and the residues Ser40-Tyr44. In contrast, the Bitistatin B has the Pro3 positioned adjacent to the side chain Glu11-Gln12, where the Val4 interacts with the residues Gly6-Gln12. An important role of the Leu10 was described, in Bitistatin B, to form a hydrophobic core to stabilize the protein with an Asn43-Tyr44 interaction. On the other hand, Bitistatin A shows a hydrophobic interaction with the Leu36 to the Ser40-Tyr44 (Carbajo et al., 2015).

Also, these groups share some signature sequences (Table 1) from group 1 (Adams and SVMP) to group 5 (medium disintegrins), which corroborate the evolutionary idea that disintegrins derived from long to short disintegrins (Bazaa et al., 2007).

Another example is acostatin classified as group 6, a heterodimeric disintegrin isolated from the venom of *Agkistrodon contortrix contortrix*. The crystal structure shows a tetramer (dimer of dimer), with each dimer with a similar fold. The structures present the same disulfide pattern of group 6, with Cys residues DJ, GI, HM, and KN forming intrachain disulfide bridges and E and F interchain disulfide bridges. Both dimers form an identical disulfide pattern. (Figure 4) (Moiseeva et al., 2008). Interestingly, if only the sequence were to be taken into account, as in the cladogram construction, chain α would be wrongly classified as group 4 and chain β as group 5 (Figure 5).

One of the most different structures reported so far is the Salmosin (group 5). This disintegrin isolated from the *Agkistrodon halys* venom has the RGD motif conserved with an unusual finger shape and is distal from the rigid nucleus of the C terminal domain. In addition, although the RGD motif does not interact with the hydrophobic nucleus of the protein, it has been stabilized by a network of molecular contacts through a small beta antiparallel sheet comprising residues of Ile46-Ala50 and Asp54-Tyr58. The distribution of electrostatic charge on the surface of the salmosin differs dramatically, in comparison with other disintegrins, showing a cluster of negatively charged residues near the RGD loop (Shin et al., 2003). NMR data further indicated that salmosin has a topology similar to kistrin (member of group 4D), although the two molecules have entirely different disulfide bond patterns. Furthermore, it has been shown that salmosin is also made up of several closed folds and irregular loops, including residues Gly3-Gly9, Cys15-Cys21, Lys22-Lys27, Leu33-Leu38, Gly44-Ile46, and Gly62-Gly65, and that these loops are stabilized by a disulfide bond matrix across Cys AD, C, BE, FI, HJ, GM, L and KN (Shin et al., 2003).

## Integrins

**FIGURE 6:**
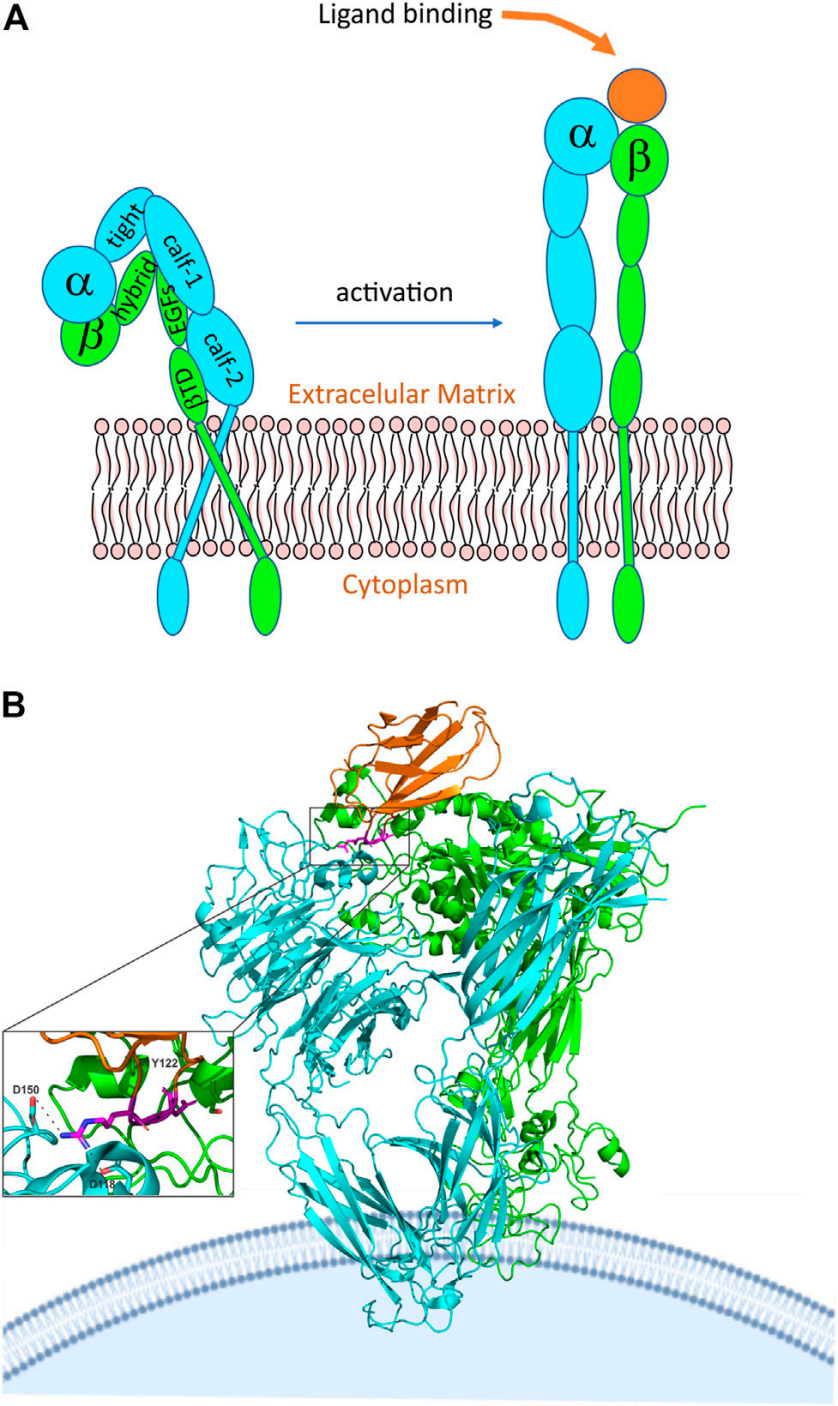
Integrin structure and activation. **(A)** General representation of integrin in the inactive closed state and the active ligand-bound state. The subunit domains are highlighted, where: EGF, epidermal growth factor; βTD, beta-tail domain. **(B)** crystal structure of alphaVbeta3 integrin interacting with fibronectin. The chains are represented by different colors, in which cyan we have the alpha chain, in green, we have the beta chain and in orange we have fibronectin. We can also see more broadly that the RGD domain (highlighted in magenta) of fibronectin is involved in the interaction with the integrin alphaVbeta3 (PDB 4MMX). The RGD motif binds to the head of αVβ3, in this interaction Tyr122 of the β subunit is important and is also conserved in other integrins, such as α5β1 and β2 integrins, which, like αVβ3, are drug targets (Goodman and Picard, 2012; Nagae et al., 2012). In addition, Asp 118 and 150 also participate in the interaction.

Integrins are a large family of surface receptors, involved in cell-cell and cell interactions with extracellular matrix components, present in biological processes such as angiogenesis and hemostasis (Hynes, 1992; Ata and Antonescu, 2017). Integrins are heterodimers formed by one α and one β-subunit, stabilized by noncovalent bonds (Barczyk et al., 2010) (Figure 6A). Each integrin subunit combines to form 24 heterodimers, composed of 18 α subunits and 8 β (Barczyk et al., 2010). Each subunit has a transmembrane polypeptide type I; containing three domains, a glycosylated domain, a hydrophobic domain, and an endo-domain. The α subunit can vary in the range of 120–180 Kda. At the N-terminus, the α subunit possesses seven homologous domains of 50 amino acids each. The extracellular portion of α subunit is composed of two calf domains, one thigh domain, one β-helix domain (β-propeller), and one ligand-binding I domain (interactive domain) named αI (Shattil and Newman, 2004; Barczyk et al., 2010; Dermont et al., 2010). β-subunit varies from 95 to 117 Kda. Each β-subunit contains a divalent cation binding site located at 100 residues from the amino terminus (Berman et al., 2003). The extracellular portion of the β subunit has four EGF-like domains, one type I domain inside in the hybrid domain that composes the integrin head, known as βI domain (Dermont et al., 2010; Coller, 2015). αI and βI domains possess specific binding regions for metallic ions. The presence of magnesium ion in the domain I of αI, known as metal ion dependent-adhesion site (MIDAS), modulates the binding of integrin to the specific ligand. Some integrins, such as αIIbβ3, lacks αI domain, in that case, the ligand-binding lies between βI domain and β-propeller of the α subunit (Dermont et al., 2010; Coller, 2015). Ligand-binding by αI and βI domains promotes a conformational change in the extracellular integrin portion, promoting separation of transmembrane and cytoplasmic portion, a process known as outside-in activation. Intracellular signaling promotes conformational changes in the cytoplasmatic integrin tail until the ligand-binding domain, known as inside-out activation (Barczyk et al., 2010; Dermont et al., 2010). Three conformational states are reported for integrin receptors: a low-affinity state, where the integrin structure is bent (Figure 6A); an intermediate affinity state triggered by the binding to the ligand, where the integrin structure is elongated, however partially activated; and a high affinity conformational, also triggered by the ligand-binding, where the integrin structure is extended and opened (Figure 6A) (Barczyk et al., 2010; Dermont et al., 2010). Structural studies on integrins have been solved mainly by x-ray crystallography of the extracellular structure fragments, αI domain, transmembrane and cytosolic complex, and intracellular protein in complex with the cytosolic tail of integrins (Liddington, 2014). Although few examples of structural experiments of integrin were reported, some integrins described on disintegrin structure studies, such as αIIbβ3 and αvβ3, have been characterized.

In addition to structural characteristics of αIIbβ3 integrin, crystallography study of integrin αvβ3 reveals that NH2-terminal segments of the α and β subunits consist of an ovoid head with two parallel tails. The head is formed by a seven-bladed β-propeller from αv and a βA domain. Also, four solvent-exposed Ca^2+^ binding sites are found in the A-B β hairpin loops of blades 4 to seven at the propeller´s bottom. Finally, the αv ends in the thigh and calf-1 and 2 domains. Each calf domain contains two antiparallel β sheets, one with four strands and the other one with five (Xiong et al., 2001). Binding to RGD peptide, reveals that the pentagonal peptide fits into a crevice between the propeller and the βA domains on the integrin head. Also, the binding is associated with tertiary and quaternary changes in the integrin; affecting the α-1 α-2 loops and helices and the α2-C, F-α7 loops. At the same time, the βA and the αv propeller suffer a small change, with the two domains moving closer together at the peptide-binding site (Xiong et al., 2001). A study of the crystal structure of integrin αvβ3 with a cyclic pentapeptide ligand RGD showed that the peptide fits in a crevice between the β-propeller and the βA domains on the integrin head. Also, this study reveals that the Arg side chain gets into a narrow groove at the top of the propeller domain. Furthermore, the Asp made contact with the βA involving the Asp carboxylate group, this made a kind of network of polar interactions, making the Asp side chain interact primarily with the βA residues. On the other hand, the Gly residue makes contact in an interface between the α and the β, making several hydrophobic interactions with the αV. The fact that the Asp interacts with the βA suggests this residue is responsible for the recognition of the βA (Xiong et al., 2001). Figure 6B shows the interaction of αvβ3 integrin with the RGD domain of fibronectin, representing the fit of the protein into the domains of the integrin. Possibly, disintegrins interact similarly, but a high-resolution structure of the disintegrin/integrin complex is not available.

## Structural Features of Disintegrin-Integrin Interaction

Only a very few reports show the interaction between disintegrin and integrins. The recognition of Integrins by disintegrins is mediated by the specific binding loop of each disintegrin, some of them containing the RGD sequence. In that matter, it is of interest to look upon the interaction of peptides containing RGD sequence and integrins.

### Functional Diversification of Disintegrins Motifs and Bases of Integrin-Modulation

Functionally, the evolution of the disintegrin family is influenced by positive Darwinian selection, guiding the adaptation of the conformation loop and C-terminal to the target integrin receptor (Juárez et al., 2008). The evolution of integrin-binding motif on disintegrins emerges from an ancestral Arg-Gly-Asp (RGD) sequence, according to phylogenetic and codon substitution studies, to a panel of integrin receptors targets (Juárez et al., 2008). RGD sequence emerged from a subgroup of PIII-SVMP with ^66^RDECD^70^ sequence. Minimum mutations accomplish the conversion of RDE to RGD sequence, and three mutations (DNA) were the minimum codon changes required to the emergence of inhibitory integrin motifs (Juárez et al., 2008). Dimeric disintegrins present different integrin-binding motifs, suggesting fast evolution and cumulative structure changes (Calvete et al., 2003). Disintegrin inhibitory loop motifs are represented by RGD, capable of modulating integrins such as αIIbβ3, αvβ3, and α5β1. Variations of RGD motif include KGD, VGD, MGD, and WGD. KTS and RTS selectively modulate α1β1; MLD motifs, capable of modulating integrins such as α9β1, α4β1 and α4β7 (Table 2); (Sanz et al., 2006). Most RGD-disintegrins are monomeric proteins and this motif is present in all homo-dimeric and some subunits of hetero-dimers disintegrins (Walsh and Marcinkiewicz, 2011).

Different structural features of disintegrins define the interaction with the integrin receptors. Conserved aspartate within the disintegrin motif might be responsible for binding to integrin receptors, while the specificity is ruled by the other two residues within the disintegrin motif. Further, the residues flanking the tripeptide motif, the conformation of the motif loop, and the C-terminal of the disintegrin sequence defined the integrin-binding and selectivity (Calvete, 2005a). This characteristic modulates the selectivity of disintegrins to different integrins, as represented in Table 3, which shows the selectivity interactions of disintegrins, divided according to each group of our classification, through different receptors, indicating the affinity of each member.

**TABLE 3:** Affinity and interactions with integrins to each group of disintegrins according to the classification proposed.

Groups	Integrins																REF
	Disintegrin	β3			β1								β 6	β7	β5	β2	
		αIIb	αv	α2	α5	α6	α3	α4	α9	α1	α2	α7	αv	α4b	αv	αM	
G 1	VAP1	—	—	No	Yes	Yes	Yes	No	—	—	—	—	—	—	—	—	Araki et al. (2002)
	SVMP Elapid (atragin)	Yes	Yes	—	No	—	—	—	—	—	—	—	—	—	—	—	Wu et al. (2016)
	Factor X activator (Russells viper venom factor X activator RVV-X)	Yes	—	—	—	—	—	—	—	—	—	—	—	—	—	—	Takeya et al. (1992)
	ADAM 22	—	Yes	No*		Yes	No*	No*	Yes	—	No*	—	—	—	—	—	D’Abaco et al. (2006)
	ADAM 10	—	—	—	Yes	—	—	—	No	—	—	—	Yes	—	—	—	Eto et al. (2002); Jones et al. (2013); Siney et al. (2017)
G 2/3	Bitistatin	Yes	Yes	—	—	—	—	—	—	—	—	—	—	—	—	—	Calvete et al. (1994); Pfaff et al. (1994); Mcquade et al. (2004)
G 4	Kistrin	Yes	Yes		No	—	—	—	—	—	—	—	—	—	—	—	Juliano et al. (1996); Dennis et al. (1990); Tselepis et al. (1997)
	Rhodostomin	Yes	Yes	—	No	—	No	—	—	—	No	—	—	—	No		Chang and Lo (1998); Yeh et al. (2001); Yang et al. (2005)
	Flavoridin	Yes	Yes	—	—	—	—	—	—	—	—	—	—	—	—	—	Pfaff et al. (1994)
	Tzabcanin	—	Yes	—	—	—	—	—	—	—	—	—	—	—	—	—	Saviola et al. (2016)
	Barbourin	Yes	No	—	No	—	—	—	—	—	—	—	—	—	—	—	Pfaff et al. (1994)
	Cerastin	Yes	Yes	—	—	—	—	—	—	—	—	—	—	—	—	—	Scarborough et al. (1991)
	Tergeminin	Yes	Yes	—	No	—	—	—	—	—	—	—	—	—	—	—	Scarborough et al. (1993)
	Jararacin	Yes	Yes	—	—	—	—	—	—	—	—	—	—	—	—	—	Scarborough et al. (1993)
	Lutosin	Yes	Yes	—	—	—	—	—	—	—	—	—	—	—	—	—	Scarborough et al. (1993)
	Viridin	Yes	Yes	—	—	—	—	—	—	—	—	—	—	—	—	—	Scarborough et al. (1993)
	Molossin	Yes	Yes	—	—	—	—	—	—	—	—	—	—	—	—	—	Scarborough et al. (1993)
	Triflavin	Yes	Yes	—	Yes	—	—	—	—	—	—	—	—	—	—	—	Sheu et al. (1996); Kaku et al. (1997)
		Cotiarin	Yes	Yes	—	—	—	—	—	—	—	—	—	—	—	—	—	Scarborough et al. (1993)
G 5	Salmosin	Yes	Yes	—	—	—	—	—	—	—	—	—	—	—	—	—	Park et al. (1998); Kang et al. (1999); Marcinkiewicz et al. (2003)
	Adinbitor	Yes	—	—	—	—	—	—	—	—	—	—	—	—	—	—	Tur-Fu et al. (1991)
	Jarastatin	Yes	—	—	—	—	—	—	—	—	—	—	—	—	—	Yes	Coelho et al. (2004); Wermelinger et al. (2009)
	Saxatilin	Yes	—	—	—	—	—	—	—	No	No	—	—	—	—	—	Hong et al. (2002); Sohn et al. (2008)
	Albolabrin	Yes	—	—	—	—	—	—	—	—	—	—	—	—	—	—	Lu et al. (1994)
G 6	Disintegrin VLO4	—	—	Yes	Yes	No	—	—	—	No	No	—	—	—	—	—	Calvete et al. (2003)
	Labein	Yes	—	—	Yes	Yes	Yes	—	—	—	—	Yes	—	—	—	—	Gasmi et al. (2001); Eble et al. (2003); Kusuma et al. (2012)
	Disintegrin VB7A	—	—	Yes	Yes	No	—	—	—	No	No	—	—	—	—	—	Calvete et al. (2003)
	Acostatin	Yes	—	—	—	—	—	—	—	—	—	—	—	—	—	—	Okuda et al. (2002)
	Schistatin	Yes	Yes	—	—	—	—	—	—	—	—	—	—	—	—	—	Okuda et al. (2001)
	disintegrin from Saw-scaled viper	Yes	Yes	—	—	—	—	Yes	—	—	—	—	—	Yes	—	—	Okuda et al. (2001)
	Disintegrin EO4A	—	—	Yes	Yes	No	Yes	—	—	No	No	—	—	—	—	—	Calvete et al. (2003)
G 7A	Labestatin	—	—	—	—	—	—	—	—	Yes	—	—	—	—	—	—	Olfa et al. (2005)
	Viperistatin	—	—	—	—	—	—	—	—	Yes	Yes	—	—	—	—	—	Staniszewska et al. (2009); Bazan-Socha et al. (2014)
	Jerdostatin	—	—	—	—	—	—	—	—	Yes	—	—	—	—	—	—	Sanz et al. (2005)
	Obustatin	—	—	—	—	—	—	—	—	Yes	—	—	—	—	—	—	Marcinkiewicz et al. (2003); Calvete et al. (2007); Ghazaryan et al. (2015)
G 7B	Multisquamatin	Yes	—	—	—	—	—	—	—	—	—	—	—	—	—	—	Trikha et al. (1994); Okuda et al. (2001)
	Echistatin	Yes	Yes		Yes	—	—	—	—	—	Yes	—	—	—	—	—	McLane et al. (1996); Surazyński et al. (2005); Szabo et al. (2012); Jing and Sun (2019)

Yes – disintegrin interact with integrin, No – disintegrin not interact with integrin, * – not interact with the alpha portion.

Moreover, the modulatory activity of disintegrins depends on the appropriate pairing of cysteine residues, contributing to the conformation of the disintegrin structure (Niewiarowski et al., 1994). The conservative aspect of cysteine residues and disulfide bond pattern among disintegrins contribute to the hypothesis of strong selection to maintain the active conformation of these proteins (Juárez et al., 2008).

### Studies of the Structure-activity Relationship

Integrins recognize many physiological ligands, including soluble and surface proteins. X-ray crystallographic structures of the extracellular domains of αVβ3 have provided insights into the integrin structure-function relationship (Xiong et al., 2002; Van Agthoven et al., 2014). Disintegrins containing RGD or KGD motifs have been reported as unique and potentially useful tools to investigate integrin-ligand interactions. This is because these motifs serve as an integrin ligand-binding site, through which it plays a key role in interacting with integrin receptors. However, these studies are still scarce, for example, there is no report of a structure obtained by crystallography of a disintegrin-integrin complex, most of the studies were conducted by docking analysis.

In general, the RGD motif and the C-terminal of disintegrins have been studied to modulate the Integrin-Disintegrin interaction. To illustrate, the molecular docking of rhodostomin into Integrin αIIbβ3, reveal a series of interactions between the Arg residue of the disintegrin with Asp 224 of the Integrin by salt bridges and with Tyr189 and Ser255 by hydrogen bonds. Also, the Asp residue of the RGD loop demonstrates to interact with the Ser123 of the β3 subunit (Chang et al., 2017). Likewise, the C-terminus of echistatin showed to interact with integrin αvβ3. The molecular docking of the echistatin into the integrin αvβ3 showed that the M28 of the side chain may interact with the D126 of the β3 subunit and the H44 of the C-terminus and the K45 of the C-terminus may interact with the side chain of M180 of the β3 subunit (Chen et al., 2020). In this regard, variations in the C-terminal region of disintegrins may be a requirement for the recognition of different integrins. Otherwise, the hydrophobic residues of flavoridin and kistrin dictate the specificity for the αIIbβ3 integrin. Also, analyses of the trimestatin reveal that the residues Pro53 and Trp67 may be important for the recognition of the β3 subunit (Bilgrami et al., 2004).

Up to appoint, the interactions between the RGD loop and the C-terminus of the disintegrins play a key role in integrin recognition. Flavoridin shows the contact of the RGD loop and the C terminal domains, involved between the residues Cys27, Ala28, Asp29 and residues Gly7, Asn11, Cys13, Leu21 and the other between Cys13, Ala25, and Cys26. Further, the studies of Senn 1993 found a connection of the C-terminal of the molecule in the residues Cys64 to Trp67 to the loop containing the RGD sequence, which suggests a role of the C-terminal in the recognition and interaction of the disintegrin with the integrin (Senn and Werner, 1993). Many studies have shown that the residues that flank the RGD motif and in the C-terminal region of the disintegrins affect their specificities and binding affinities to integrins (Dennis et al., 1993; Marcinkiewicz et al., 1999; Chen et al., 2009; Cheng et al., 2012).

Finally, the study of the interaction of jararacin and jarastatin with αIIbβ3 integrin by docking reveals, in the first place, that jararacin has more interactions with this integrin. The RGD motif of the jararacin interacts with both subunits of the integrin, making hydrogen bonds and ionic interactions, and indicates that the N-terminus and C-terminus region interact with both subunits of the integrin too. In the same way, the docking complex of the jarastatin showed hydrogen and ionic interactions and interactions of the N-terminus and C-terminus with both parts of the integrin (Wermelinger et al., 2009).

### Mutants in the Study of the Structure-Activity Relationship

Several studies have shown that the amino acid residues that flank the RGD motif and the C-terminal region of the disintegrins modulate their specificity of interaction with integrin complexes (Dennis et al., 1993; Scarborough et al., 1993; McLane et al., 1998; Rahman et al., 1998; Cheng et al., 2012; Shiu et al., 2012). For example, disintegrins that have an ARGDW sequence showed a greater affinity for binding to the αIIbβ3 integrin, while disintegrins with an ARGDN sequence preferentially bind to αvβ3 and α5β1 integrins (Scarborough et al., 1993). To a better understanding of these interactions, some mutants were produced over the years. To investigate the structural basis of the integrin inhibitory potency, some studies have made mutations in the amino acid residues of the interaction loop of the disintegrins and analyzed the effects produced on the structure and activity of the disintegrins by replacing the native motif (Chen et al., 2009; Carbajo et al., 2011; Calvanese et al., 2015; Chang et al., 2017). These studies have revealed that mutations in the disintegrin that change the aspartate to glutamate, in the RGD loop, decrease their activity, as for Kistrin, which showed a 100-fold decrease in activity with this mutation (Dennis et al., 1993). Likewise, the rhodostomin (Rho) D51E mutant (2PJG/2PJF) was 1,000 times less active than Rho in inhibiting integrins.

A powerful tool to investigate this is NMR spectroscopy and molecular docking has been used as valuable tools to study the relationship between the structure, dynamics, and function of the mutant strains of the rhodostomin protein (2PJF) (Table 2) to understand the important structural requirements for the recognition of integrins. The structural study carried out by Chen et al. (2009), found that Rho and its mutant have the same tertiary fold with three double-stranded antiparallel beta-sheets. There are no structural differences between the RG [D/E] loop. Two small differences between Rho and its mutant D51E were found only in its backbone dynamics and 3D structures. The relaxation parameter R_2_ value of E51 is 13% higher than that of residue D51. A difference in charge separation of 1.76 A was found between the positive (R49) and negative (D51 or E51) side chains.

The coupling of Rho to the αvβ3 integrin, by molecular docking, showed that the amide and carbonyl groups of the main structure of the amino acid D51 of Rho formed hydrogen bonds with the amino acids R216 and R214 of the integrin. However, this hydrogen bond does not exist in the structure of the complex formed by the mutant protein D51E and the integrin. Therefore, the study suggests that the hydrogen bonds between the side chain and the backbone of the D51 residue of Rho and integrin are important for their binding to integrin (Chen et al., 2009). In addition, the Rho mutant P48A (2PJI) has its inhibitory capacity to αvβ1 integrin increased by 4.4 times. Docking of P48A showed no difference in the structure of the complex with α5β1 integrin, pointing out the importance of the dynamics, especially of the RGD loop. The mutant P48A caused differences in the order parameter (S^2^), conformational exchange contribution (R_ex_), and local correlation time (τ_e_). The authors showed that the thermal flexibility (S^2^), which are motions in the pico to nanoseconds timescale, are increased for residues R49, G50, and D51 in the mutant P48A (Shiu et al., 2012).

The Rho mutant G50L (2LJV) is a disintegrin that specifically binds to αvβ3 integrin. According to Shiu et al. (2012), the docking models of the mutant G50L and integrin showed that the amino acid L50 mutant G50L can be accommodated by a cavity within the interface between the αv and β3 subunits of the αvβ3 integrin. In contrast, such a pocket is not found in αIIbβ3 integrin, due to the formation of hydrogen bonds between αIIb residue Y190 and residue R216 of β3 subunits, resulting in blocking the bonding of residue L50. It was also observed that the G50L mutation increased the rigidity of the RLD motif, and the adjacent residues exhibited a slow conformational exchange. This finding shows that the slow movements of the RLD motif also play a vital role in modulating the integrin recognition link (Chuang et al., 2012; Shiu et al., 2012).

The ARLDDL mutant (3UCI), a potent and selective αvβ3 integrin antagonist, was designed to investigate the function-structure-dynamic relationship. The 3D structure of the ARLDDL mutant was determined by X-ray crystallography, and its tertiary fold is the same as the reported disintegrin structures. The only difference found in the RLD motif of the ARLDDL loop was a compact β-turn structure with a distance of 5.5 Å between R49 (Cα) and D52 (Cα) compared to those of disintegrins ranging from 6.8 to 8.4 Å (Shiu, 2011; Shiu et al., 2012).

In the study by Chang et al. (2017), it was observed that the content of the sequence of the RGD loop and the C terminal of the disintegrins mutually affected their conformations, resulting in functional and structural differences in the integration of the integrin. Structural analysis by NMR showed that Rho mutants containing a ^48^ARGDWN-^65^PRNPWNG sequence exhibited the highest selectivity in inhibiting cell adhesion mediated by αIIbβ3 integrin. The results, of molecular docking, suggested that the content of the sequence and the length of the C-terminal regions in the disintegrins are critical to their ability to bind to the αIIbβ3 integrin (Chang et al., 2017).

Carbajo et al. (2011) made changes to the structure of jerdostatin by replacing the native RTS motif with KTS. These authors demonstrated by NMR that wild-type jerdostatin and its mutant R24K present a common structure, but different dynamic profiles. They found differences in movements on the picosecond to nanosecond time scale and deceleration movements for some residues of the R24K mutant compared to the wild type of jerdostatin (Carbajo et al., 2011). According to the authors, these findings may explain the reduction in the inhibitory potency of the integrin of the mutant jerdostatin R24K (IC_50_ 703 nM) compared to the wild type (IC_50_ 180 nM) (Carbajo et al., 2011). A complementary study by Calvanese et al. (2015), used Molecular Dynamics (MD) simulations of the two molecules (wild type jerdostatin and it is mutant) to explore in atomic resolution the structural bases of their different dynamic behaviors, to identify the atomic movements that could differentiate their behaviors dynamic and, therefore, their properties/activities. The analysis confirmed the combined movements between the recognition loop and the C-terminal tail considered relevant to the functional capacity of jerdostatin. It also revealed the residues that dominate such a mechanism. Indicating that both wild-type jerdostatin and the R24K mutant share a common structure but differ in global movements. Studies like this are important to clarify whether disintegrins movements can be functional for integrin binding (Sanz-Soler et al., 2012; Calvanese et al., 2015).

This kind of study helped the development of pharmacological agents that block platelet aggregation by inhibition of integrin αIIbβ3. Some commercialized drugs are the eptifibatide, a cyclic heptapeptide that originated from the disintegrin barbourin, and the tirofiban, which originated from the disintegrin echistatin. The barbourin is a disintegrin isolated from the venom of *Sistrurus miliarius barbouri*, which possesses a KGD loop and a high affinity to integrin αIIbβ3 (Tcheng and O’Shea, 2002). Jing and Lu 2005, investigated a mutant proinsulin chimera with eight amino acids from barbourin (CAKGDWNC, respectively). Interestingly, they found that the protein inhibits human platelet aggregation induced by ADP and retains its binding activity to the insulin receptor. Also, Xiao et al., 2004, showed how the eptifibatide fits into the Integrin αIIbβ3, demonstrating some hydrophobic contacts with the Lys, based on the binding loop of barbourin, with Phe231 in the α_IIb_ β-propeller. In addition, the Asp224 of the residue αIIb may form hydrogen bonds with the eptifibatide.

Otherwise, interaction studies between tirofiban (based on echistatin) showed that the co-crystal between the drug and the Integrin αIIbβ3 reveals that the sulfonamide groups (in the tirofiban) interacted with Tyr166 and Arg214 by hydrogen bonds in the β3 subunit and the butyl and pyridyl groups may interact with the Phe160 and Tyr190 in the αIIb subunit by hydrogen bond interaction (Xiao et al., 2004). Interestingly, the interaction with free integrin reveals poor changes in integrin αIIbβ3 structure, changing the globular conformation of the free Integrin to an open conformation in just 4% of the free integrins, seen by transmission electron microscopy (Hantgan, et al., 2002).

## Conclusion and Future Directions

To conclude, disintegrins are divided into five different groups according to their polypeptide length and the number of disulfide bonds. In this work, we propose a classification based on patterns of disulfide bonds, where this classification resulted in the division into seven groups, organized by disulfide bind pattern. Through comparing amino acid sequences by multiple-sequence alignment, a brief phylogenetic analysis, and extensive literature review, we support the view that the different disintegrin subfamilies evolved from a common ADAM (a disintegrin and metalloproteinase-like) and that structural diversification occurred through disulfide bond engineering.

A deep analysis of the conserved cysteine residues in each disintegrin subfamily (Figure 4) strongly indicates that structural diversity of disintegrins was achieved during evolution through selective loss of disulfide bonds. It is well-known that disulfide bonds play a key role in the stability and impose distinct protein folding, with a specific orientation of the loop regions of these proteins. Recognition of integrins by disintegrins is mediated by the specific binding loop of each disintegrin. As well that, the residues flanking the tripeptide motif, the motif loop conformation, and the C-terminus of the disintegrin sequence influence integrin binding and selectivity (Calvete, 2005a). The C-terminal region of disintegrins and the flexibility of the RGD loop (located at the apex of the loop), may be a requirement for the recognition of different integrins, specifically for the β subunit. These two parts mutually affected their conformations, making it crucial to the Integrin modulation. This characteristic modulates the selectivity of disintegrins to different integrins, as mentioned in this work. Furthermore, the modulatory activity of disintegrins depends on the proper pairing of cysteine residues, contributing to the conformation of the disintegrin structure (Niewiarowski et al., 1994). The conservative aspect of cysteine residues and the disulfide bond pattern between disintegrins contribute to the hypothesis of strong selection to maintain the active conformation of these proteins (Juárez et al., 2008).

Finally, few structural studies show the interaction between disintegrins and integrins. In our work, the selective interactions of disintegrins are shown, divided according to each group of our classification, through different receptors, indicating the affinity of each member to differents integrins, exhibit the importance of the structural studies to the comprehension of the interaction of the Disintegrins to its receptor targets. This knowledge is fundamental for the designing of new drugs that target integrins, as it is shown that disulfide arrangements of disintegrins have an impact on integrin/disintegrin interaction. Also, it is important to point out that only a small percentage of the available disintegrins from venoms has been investigated so far and these molecules present opportunities for larger Integrin engagement surface with good stability, increasing the opportunity for the development of new drugs (Trim et al., 2021).

## Footnotes

1 The snake species names were taken from the original papers that describe the disintegrins, some scientifics names of these species could have changed.

## References

[B1] Almeida F. C. L. Sanches K. Pinheiro-Aguiar R. Almeida V. S. Caruso I. P. (2021). Protein Surface Interactions-Theoretical and Experimental Studies. Front. Mol. Biosci. 8, 1–10. 10.3389/fmolb.2021.706002 PMC8298896 34307462

[B2] Altschul S. F. Gish W. Miller W. Myers E. W. Lipman D. J. (1990). Basic Local Alignment Search Tool. J. Mol. Biol. 215, 403–410. 10.1016/S0022-2836(05)80360-2 2231712

[B3] Araki S. Masuda S. Maeda H. Ying M. J. Hayashi H. (2002). Involvement of Specific Integrins in Apoptosis Induced by Vascular Apoptosis-Inducing Protein 1. Toxicon 40, 535–542. 10.1016/S0041-0101(01)00249-5 11821125

[B4] Arruda Macedo J. Fox J. Souza Castro M. (2015). Disintegrins from Snake Venoms and Their Applications in Cancer Research and Therapy. Curr. Protein Pept. Sci. 16, 532–548. 10.2174/1389203716666150515125002 26031306 PMC4997955

[B5] Ata R. Antonescu C. (2017). Integrins and Cell Metabolism: An Intimate Relationship Impacting Cancer. Int. J. Mol. Sci. 18, 189. 10.3390/ijms18010189 PMC5297821 28106780

[B6] Barczyk M. Carracedo S. Gullberg D. (2010). Integrins. Cell Tissue Res 339, 269–280. 10.1007/s00441-009-0834-6 19693543 PMC2784866

[B7] Bazaa A. Juárez P. Marrakchi N. Lasfer Z. B. Ayeb M. E. Harrison R. A. (2007). Loss of Introns Along the Evolutionary Diversification Pathway of Snake Venom Disintegrins Evidenced by Sequence Analysis of Genomic DNA from Macrovipera Lebetina Transmediterranea and Echis Ocellatus. J. Mol. Evol. 64, 261–271. 10.1007/s00239-006-0161-4 17177090

[B8] Bazan-Socha S. Żuk J. Plutecka H. Jakieła B. Mlicka-Kowalczyk E. Krzyżanowski B. (2014). Blocking of α1β1 and α2β1 Adhesion Molecules Inhibits Eosinophil Migration through Human Lung Microvascular Endothelial Cell Monolayer. Postepy Hig. Med. Dosw. 68, 1444–1451. 10.5604/17322693.1131696 25531708

[B9] Berman A. E. Kozlova N. I. Morozevich G. E. (2003). Integrins: Structure and Signaling. Biochemistry (Mosc) 68, 1284–1299. 10.1023/b:biry.0000011649.03634.74 14756624

[B10] Bilgrami S. Tomar S. Yadav S. Kaur P. Kumar J. Jabeen T. (2004). Crystal Structure of Schistatin, a Disintegrin Homodimer from Saw-Scaled Viper (*Echis C*) at 2.5Å Resolution. J. Mol. Biol. 341, 829–837. 10.1016/j.jmb.2004.06.048 15317139

[B11] Bilgrami S. Yadav S. Kaur P. Sharma S. Perbandt M. Betzel C. (2005). Crystal Structure of the Disintegrin Heterodimer from Saw-Scaled Viper (*Echis C*) at 1.9 Å Resolution. Biochemistry 44, 11058–11066. 10.1021/bi050849y 16101289

[B12] Blodel C. P. White J. M. (1992). Structure, Function and Evolutionary Relationship of Proteins Containing a Disintegrin Domain. Curr. Opin. Cel Biol. 4, 760–765. 10.1016/0955-0674(92)90098-W 1419054

[B13] Borges M. H. Figueiredo S. G. Leprevost F. V. De Lima M. E. Cordeiro M. d. N. Diniz M. R. V. (2016). Venomous Extract Protein Profile of Brazilian Tarantula Grammostola Iheringi : Searching for Potential Biotechnological Applications. J. Proteomics 136, 35–47. 10.1016/j.jprot.2016.01.013 26828374

[B14] Calvanese L. Falcigno L. D'Auria G. (2015). Essential Dynamics Analysis Captures the Concerted Motion of the Integrin-Binding Site in Jerdostatin, an RTS Disintegrin. Biopolymers 103, 158–166. 10.1002/bip.22578 25363370

[B15] Calvete J. J. (2010). “Brief History and Molecular Determinants of Snake Venom Disintegrin Evolution,” in Toxins and Hemostasis. Editors Kini R. Clemetson K. Markland F. McLane M. Morita T. (Dordrecht: Springer), 285–300. 10.1007/978-90-481-9295-3_18

[B16] Calvete J. J. Marcinkiewicz C. Monleón D. Esteve V. Celda B. Juárez P. (2005b). Snake Venom Disintegrins: Evolution of Structure and Function. Toxicon 45, 1063–1074. 10.1016/j.toxicon.2005.02.024 15922775

[B17] Calvete J. J. McLane M. A. Stewart G. J. Niewiarowski S. (1994). Characterization of the Cross-Linking Site of Disintegrins Albolabrin, Bitistatin, Echistatin, and Eristostatin on Isolated Human Platelet Integrin GpIIb/IIIa. Biochem. Biophysical Res. Commun. 202, 135–140. 10.1006/bbrc.1994.1903 8037704

[B18] Calvete J. J. Moreno-Murciano M. P. Sanz L. Jurgens M. Schrader M. Raida M. (2000). The Disulfide Bond Pattern of Catrocollastatin C, a Disintegrin-Like/Cysteine-Rich Protein Isolated fromCrotalus Atroxvenom. Protein Sci. 9, 1365–1373. 10.1110/ps.9.7.1365 10933502 PMC2144675

[B19] Calvete J. J. Moreno-Murciano M. P. Theakston R. D. G. Kisiel D. G. Marcinkiewicz C. (2003). Snake Venom Disintegrins: Novel Dimeric Disintegrins and Structural Diversification by Disulphide Bond Engineering. Biochem. J. 372, 725–734. 10.1042/bj20021739 12667142 PMC1223455

[B20] Calvete J. J. (2013). The Continuing Saga of Snake Venom Disintegrins. Toxicon 62, 40–49. 10.1016/j.toxicon.2012.09.005 23010163

[B21] Calvete J. Marcinkiewicz C. Sanz L. (2007). KTS and RTS-Disintegrins: Anti-Angiogenic Viper Venom Peptides Specifically Targeting the α1β 1 Integrin. Curr. Pharm. Des. 13, 2853–2859. 10.2174/138161207782023766 17979730

[B22] Calvete J. (2005a). Structure-Function Correlations of Snake Venom Disintegrins. Curr. Pharm. Des. 11, 829–835. 10.2174/1381612053381783 15777237

[B23] Carbajo R. J. Sanz L. Mosulén S. Pérez A. Marcinkiewicz C. Pineda-Lucena A. (2011). NMR Structure and Dynamics of Recombinant Wild Type and Mutated Jerdostatin, a Selective Inhibitor of Integrin α1 β1. Proteins 79, 2530–2542. 10.1002/prot.23076 21656569

[B24] Carbajo R. J. Sanz L. Perez A. Calvete J. J. (2015). NMR Structure of Bitistatin - A Missing Piece in the Evolutionary Pathway of Snake Venom Disintegrins. FEBS J. 282, 341–360. 10.1111/febs.13138 25363287

[B25] Cesar P. H. S. Braga M. A. Trento M. V. C. Menaldo D. L. Marcussi S. (2019). Snake Venom Disintegrins: An Overview of Their Interaction with Integrins. Curr. Drug Targets 20, 465–477. 10.2174/1389450119666181022154737 30360735

[B26] Chang H.-H. Lo S. J. (1998). Full-Spreading Platelets Induced by the Recombinant Rhodostomin Are via Binding to Integrins and Correlated with FAK Phosphorylation. Toxicon 36, 1087–1099. 10.1016/S0041-0101(98)00088-9 9690777

[B27] Chang Y.-T. Shiu J.-H. Huang C.-H. Chen Y.-C. Chen C.-Y. Chang Y.-S. (2017). Effects of the RGD Loop and C-Terminus of Rhodostomin on Regulating Integrin αIIbβ3 Recognition. PLoS One 12, e0175321. 10.1371/journal.pone.0175321 28399159 PMC5388508

[B28] Chang Y. Jeng W. Shiu J. Chang C. Chuang W. (2014). Crystal Structure of Rhodostomin ARGDP Mutant. Available at: https://www.rcsb.org/structure/4M4C (Accessed June 15, 2021). 10.2210/pdb4m4c/pdb

[B29] Chen C.-Y. Shiu J.-H. Hsieh Y.-H. Liu Y.-C. Chen Y.-C. Chen Y.-C. (2009). Effect of D to E Mutation of the RGD Motif in Rhodostomin on its Activity, Structure, and Dynamics: Importance of the Interactions between the D Residue and Integrin. Proteins 76, 808–821. 10.1002/prot.22387 19280603

[B30] Chen Y.-C. Chang Y.-T. Chen C.-Y. Shiu J.-H. Cheng C.-H. Huang C.-H. (2020). Structural Insight into Integrin Recognition and Anticancer Activity of Echistatin. Toxins 12, 709. 10.3390/toxins12110709 PMC7695343 33182321

[B31] Chen Y. Pitzenberger S. M. Garsky V. M. Lumma P. K. Sanyal G. Baum J. (1991). Proton NMR Assignments and Secondary Structure of the Snake Venom Protein Echistatin. Biochemistry 30, 11625–11636. 10.1021/bi00114a004 1661142

[B32] Cheng C.-H. Chen Y.-C. Shiu J.-H. Chang Y.-T. Chang Y.-S. Huang C.-H. (2012). Dynamics and Functional Differences between Dendroaspin and Rhodostomin: Insights into Protein Scaffolds in Integrin Recognition. Protein Sci. 21, 1872–1884. 10.1002/pro.2169 23033223 PMC3575917

[B33] Chuang W. Chang Y. . Shiu J. Chen C. Chen Y. (2013). The C-Terminal Region of Disintegrin Modulate its 3D Conformation and Cooperate with RGD Loop in Regulating Recognitions of Integrins. Available at: https://www.rcsb.org/structure/2M7H (Accessed June 15, 2021). 10.2210/pdb2m75/pdb

[B34] Chuang W. Chen C. Shiu J. Chen Y. (2004). Structural Analysis of Integrin Alpha IIb Beta 3- Disintegrin with the AKGDWN Moti. Available at: https://www.rcsb.org/structure/1Q7I (Accessed June 15, 2021).

[B35] Chuang W. Liu Y. Shiu J. (2007). Solution Structure of Rhodostomin P48A Mutant. Available at: https://www.rcsb.org/structure/2PJI (Accessed June 15, 2021). 10.2210/pdb2pji/pdb

[B36] Chuang W. Shiu J. Chen C. Chen Y. Huang C. (2012). Solution Structure of Rhodostomin G50L Mutant. Available at: https://www.rcsb.org/structure/2LJV (Accessed June 15, 2021). 10.2210/pdb2ljv/pdb

[B37] Cidade D. A. P. Wermelinger L. S. Lôbo-Hajdu G. Dávila A. M. R. Bon C. Zingali R. B. (2006). Molecular Diversity of Disintegrin-Like Domains within Metalloproteinase Precursors of *Bothrops Jararaca* . Toxicon 48, 590–599. 10.1016/j.toxicon.2006.07.010 16919699

[B38] Coelho A. L. J. De Freitas M. S. Mariano-Oliveira A. Rapozo D. C. M. Pinto L. F. R. Niewiarowski S. (2004). RGD- and MLD-Disintegrins, Jarastatin and EC3, Activate Integrin-Mediated Signaling Modulating the Human Neutrophils Chemotaxis, Apoptosis and IL-8 Gene Expression. Exp. Cel Res. 292, 371–384. 10.1016/j.yexcr.2003.09.013 14697344

[B39] Coelho A. L. J. De Freitas M. S. Oliveira-Carvalho A. L. Moura-Neto V. Zingali R. B. Barja-Fidalgo C. (1999). Effects of Jarastatin, a Novel Snake Venom Disintegrin, on Neutrophil Migration and Actin Cytoskeleton Dynamics. Exp. Cel Res. 251, 379–387. 10.1006/excr.1999.4583 10471323

[B40] Coller B. S. (2015). αIIbβ3: Structure and Function. J. Thromb. Haemost. 13, S17–S25. 10.1111/jth.12915 26149019 PMC4888797

[B41] Cooke R. M. Carter B. G. Murray-Rust P. Hartshorn M. J. Herzyk P. Hubbard R. E. (1992). The Solution Structure of Echistatin: Evidence for Disulphide Bond Rearrangement in Homologous Snake Toxins. Protein Eng. Des. Sel. 5, 473–477. 10.1093/protein/5.6.473 1438157

[B42] D'Abaco G. M. Ng K. Paradiso L. Godde N. J. Kaye A. Novak U. (2006). ADAM22, Expressed in normal Brain but Not in High-Grade Gliomas, Inhibits Cellular Proliferation via the Disintegrin Domain. Neurosurgery 58, 179–186. 10.1227/01.NEU.0000192363.84287.8B 16385342

[B43] David V. Succar B. B. de Moraes J. A. Saldanha-gama R. F. G. Barja-fidalgo C. Zingali R. B. (2018). Recombinant and Chimeric Disintegrins in Preclinical Research. Toxins 10, 321–324. 10.3390/toxins10080321 PMC6116119 30087285

[B44] Dennis M. S. Carter P. Lazarus R. A. (1993). Binding Interactions of Kistrin with Platelet Glycoprotein IIb-IIIa: Analysis by Site-Directed Mutagenesis. Proteins 15, 312–321. 10.1002/prot.340150308 8456099

[B45] Dennis M. S. Henzel W. J. Pitti R. M. Lipari M. T. Napier M. A. Deisher T. A. (1990). Platelet Glycoprotein IIb-IIIa Protein Antagonists from Snake Venoms: Evidence for a Family of Platelet-Aggregation Inhibitors. Proc. Natl. Acad. Sci. 87, 2471–2475. 10.1073/pnas.87.7.2471 2320569 PMC53711

[B46] Dermont C. Brennan M. Moran N. (2010). Integrins as Therapeutic Targets: Lessons and Opportunities. Nat. Rev. Drug Discov. 9, 804–820. 10.1038/nrd3266 20885411

[B47] Eble J. A. Bruckner P. Mayer U. (2003). Vipera Lebetina Venom Contains Two Disintegrins Inhibiting Laminin-Binding β1 Integrins. J. Biol. Chem. 278, 26488–26496. 10.1074/jbc.M301860200 12719418

[B48] Eto K. Huet C. Tarui T. Kupriyanov S. Liu H.-Z. Puzon-McLaughlin W. (2002). Functional Classification of ADAMs Based on a Conserved Motif for Binding to Integrin α9β1. J. Biol. Chem. 277, 17804–17810. 10.1074/jbc.M200086200 11882657

[B49] Fox J. W. Serrano S. M. T. (2009). Timeline of Key Events in Snake Venom Metalloproteinase Research. J. Proteomics 72, 200–209. 10.1016/j.jprot.2009.01.015 19344655

[B50] Fujii Y. Okuda D. Fujimoto Z. Horii K. Morita T. Mizuno H. (2003). Crystal Structure of Trimestatin, a Disintegrin Containing a Cell Adhesion Recognition Motif RGD. J. Mol. Biol. 332, 1115–1122. 10.1016/S0022-2836(03)00991-4 14499613

[B51] Gasmi A. Srairi N. Guermazi S. Dkhil H. Karoui H. El Ayeb M. (2001). Amino Acid Structure and Characterization of a Heterodimeric Disintegrin from Vipera Lebetina Venom. Biochim. Biophys. Acta (Bba) - Protein Struct. Mol. Enzymol. 1547, 51–56. 10.1016/S0167-4838(01)00168-6 11343790

[B52] Ghazaryan N. A. Ghulikyan L. A. Kishmiryan A. V. Kirakosyan G. R. Nazaryan O. H. Ghevondyan T. H. (2015). Anti-tumor Effect Investigation of Obtustatin and Crude *Macrovipera L* Obtusa Venom in S-180 Sarcoma Bearing Mice. Eur. J. Pharmacol. 764, 340–345. 10.1016/j.ejphar.2015.07.011 26169565

[B53] Gish W. States D. J. (1993). Identification of Protein Coding Regions by Database Similarity Search. Nat. Genet. 3, 266–272. 10.1089/cmb.1994.1.3910.1038/ng0393-266 8485583

[B54] Goodman S. L. Picard M. (2012). Integrins as Therapeutic Targets. Trends Pharmacol. Sci. 33, 405–412. 10.1016/j.tips.2012.04.002 22633092

[B55] Gould R. J. Polokoff M. A. Friedman P. A. Huang T.-F. Holt J. C. Cook J. J. (1990). Disintegrins: A Family of Integrin Inhibitory Proteins from Viper Venoms. Exp. Biol. Med. 195, 168–171. 10.3181/00379727-195-43129b 2236100

[B56] Guo R.-T. Chou L.-J. Chen Y.-C. Chen C.-Y. Pari K. Jen C. J. (2001). Expression inPichia Pastoris and Characterization by Circular Dichroism and NMR of Rhodostomin. Proteins 43, 499–508. 10.1002/prot.1061 11340665

[B57] Hassan M. Ethayathulla A. Bilgrami S. Singh B. Yadav S. Singh T. (2005). Crystal Structure of a Novel Disintegrin from Saw-Scaled viper at 3.2 A Resolution. Available at: https://www.rcsb.org/structure/1Z1X (Accessed June 15, 2021). 10.2210/pdb1z1x/pdb

[B58] Hong S.-Y. Koh Y.-S. Chung K.-H. Kim D.-S. (2002). Snake Venom Disintegrin, Saxatilin, Inhibits Platelet Aggregation, Human Umbilical Vein Endothelial Cell Proliferation, and Smooth Muscle Cell Migration. Thromb. Res. 105, 79–86. 10.1016/S0049-3848(01)00416-9 11864711

[B155] Huang C. H. Shiu J. Chang Y. Jeng W. Chuang W. (2015a). Crystal structure of Rhodostomin R46E mutant. Available at: https://www.rcsb.org/structure/4R5U (Accessed June 15, 2021). 10.2210/pdb4r5r/pdb

[B59] Huang C. H. Shiu J. Chang Y. Jeng W. Chuang W. (2015b). Crystal Structure of Rhodostomin KKKRT Mutant. Available at: https://www.rcsb.org/structure/4R5R. (Accessed June 15, 2021) 10.2210/pdb4r5r/pdb

[B60] Huang T.-F. Hsu C.-C. Kuo Y.-J. (2016). Anti-thrombotic Agents Derived from Snake Venom Proteins. Thromb. J 14, 18. 10.1186/s12959-016-0113-1 27766044 PMC5056499

[B61] Huang T.-F. Lukasiewicz H. Holt C. J. Niewiarowski S. (1987). “Characterization of Fibrinogen Receptors Associated with Glicoproiein IIb/IIIa (GPIIb/GPIIIa) Complex by Trigramin, a Unique Low Molecular Weight Peptide Probe,” in XIth Int. Congr. Thromb. Haemost. Stuttgart, (Accessed April 11, 2021). 10.1055/s-0038-1643523

[B62] Huang T.-F. Sheu J.-R. Teng C.-M. (1991b). Mechanism of Action of a Potent Antiplatelet Peptide, Triflavin from Trimeresurus flavoviridis Snake Venom. Thromb. Haemost. 66, 489–493. 10.1055/s-0038-1646444 1665595

[B63] Huang T. F. Sheu J. R. Teng C. M. (1991a). A Potent Antiplatelet Peptide, Triflavin, from Trimeresurus flavoviridis Snake Venom. Biochem. J. 277, 351–357. 10.1042/bj2770351 1859363 PMC1151241

[B64] Hynes R. (1987). Integrins: A Family of Cell Surface Receptors. Cell 48, 549–554. 10.1016/0092-8674(87)90233-9 3028640

[B65] Hynes R. O. (1992). Integrins: Versatility, Modulation, and Signaling in Cell Adhesion. Cell 69, 11–25. 10.1016/0092-8674(92)90115-s 1555235

[B66] Jia L. G. Shimokawa K. I. Bjarnason J. B. Fox J. W. (1996). Snake Venom Metalloproteinaes: Structure, Function and Relationship to the adams Family of Proteins. Toxicon 34, 1269–1276. 10.1016/S0041-0101(96)00108-0 9027982

[B67] Jing J. Lu S. (2005). Inhibition of Platelet Aggregation of a Mutant Proinsulin Chimera Engineered by Introduction of a Native Lys-Gly-Asp-Containing Sequence. Biotechnol. Lett. 27, 1259–1265. 10.1007/s10529-005-3202-y 16215822

[B68] Jing J. Sun Y. (2019). An αIIbβ3- and Phosphatidylserine (PS)-Binding Recombinant Fusion Protein Promotes PS-Dependent Anticoagulation and Integrin-Dependent Antithrombosis. J. Biol. Chem. 294, 6670–6684. 10.1074/jbc.RA118.006044 30803987 PMC6497940

[B69] Jones A. V. Lambert D. W. Speight P. M. Whawell S. A. (2013). ADAM 10 Is over Expressed in Oral Squamous Cell Carcinoma and Contributes to Invasive Behaviour through a Functional Association with αvβ6 Integrin. FEBS Lett. 587, 3529–3534. 10.1016/j.febslet.2013.09.010 24055471

[B70] Juárez P. Comas I. González-Candelas F. Calvete J. J. (2008). Evolution of Snake Venom Disintegrins by Positive Darwinian Selection. Mol. Biol. Evol. 25, 2391–2407. 10.1093/molbev/msn179 18701431

[B71] Juárez P. Wagstaff S. C. Oliver J. Sanz L. Harrison R. A. Calvete J. J. (2006a). Molecular Cloning of Disintegrin-Like Transcript BA-5A from a Bitis Arietans Venom Gland cDNA Library: A Putative Intermediate in the Evolution of the Long-Chain Disintegrin Bitistatin. J. Mol. Evol. 63, 142–152. 10.1007/s00239-005-0268-z 16786436

[B72] Juárez P. Wagstaff S. C. Sanz L. Harrison R. A. Calvete J. J. (2006b). Molecular Cloning of Echis Ocellatus Disintegrins Reveals Non-Venom-Secreted Proteins and a Pathway for the Evolution of Ocellatusin. J. Mol. Evol. 63, 183–193. 10.1007/s00239-005-0269-y 16830094

[B73] Juliano D. Wang Y. Marcinkiewicz C. Rosenthal L. A. Stewart G. J. Niewiarowski S. (1996). Disintegrin Interaction with αvβ3Integrin on Human Umbilical Vein Endothelial Cells: Expression of Ligand-Induced Binding Site on β3Subunit. Exp. Cel Res. 225, 132–142. 10.1006/excr.1996.0164 8635506

[B74] Kaku S. Umemura K. Mizuno A. Kawasaki T. Nakashima M. (1997). Evaluation of the Disintegrin, Triflavin, in a Rat Middle Cerebral Artery Thrombosis Model. Eur. J. Pharmacol. 321, 301–305. 10.1016/S0014-2999(96)00971-5 9085041

[B75] Kang I. C. Lee Y. D. Kim D. S. (1999). A Novel Disintegrin Salmosin Inhibits Tumor Angiogenesis. Cancer Res. 59, 3754–3760. 10446992

[B76] Khamessi O. Ben Mabrouk H. Othman H. ElFessi-Magouri R. De Waard M. Hafedh M. (2018). RK, the First Scorpion Peptide with Dual Disintegrin Activity on α1β1 and αvβ3 Integrins. Int. J. Biol. Macromolecules 120, 1777–1788. 10.1016/j.ijbiomac.2018.09.180 30287364

[B77] Kini R. M. Evans H. J. (1992). Structural Domains in Venom Proteins: Evidence that Metalloproteinases and Nonenzymatic Platelet Aggregation Inhibitors (Disintegrins) from Snake Venoms Are Derived by Proteolysis from a Common Precursor. Toxicon 30 (3), 265–293. 10.1016/0041-0101(92)90869-7 1529462

[B78] Kisiel D. G. Calvete J. J. Katzhendler J. Fertala A. Lazarovici P. Marcinkiewicz C. (2004). Structural Determinants of the Selectivity of KTS-Disintegrins for the α1β1 Integrin. FEBS Lett. 577, 478–482. 10.1016/j.febslet.2004.10.050 15556632

[B79] Kumar S. Stecher G. Li M. Knyaz C. Tamura K. (2018). Mega X: Molecular Evolutionary Genetics Analysis across Computing Platforms. Mol. Biol. Evol. 35, 1547–1549. 10.1093/molbev/msy096 29722887 PMC5967553

[B80] Kuo Y.-J. Chung C.-H. Huang T.-F. (2019). From Discovery of Snake Venom Disintegrins to a Safer Therapeutic Antithrombotic Agent. Toxins 11, 1–11. 10.3390/toxins11070372 PMC6669693 31247995

[B81] Kusuma N. Denoyer D. Eble J. A. Redvers R. P. Parker B. S. Pelzer R. (2012). Integrin-Dependent Response to Laminin-511 Regulates Breast Tumor Cell Invasion and Metastasis. Int. J. Cancer 130, 555–566. 10.1002/ijc.26018 21387294

[B82] Lazarovici P. Marcinkiewicz C. Lelkes P. I. (2019). From Snake Venom's Disintegrins and C-Type Lectins to Anti-Platelet Drugs. Toxins 11, 303–315. 10.3390/toxins11050303 PMC6563238 31137917

[B83] Liddington R. C. (2014). Structural Aspects of Integrins. Adv. Exp. Med. Biol. 819, 111–126. 10.1007/978-94-017-9153-3_8 25023171

[B84] Lima M. E. D. Pimenta A. M. D. C. Martin-Eauclaire M. F. Zingali R. B. Rochat H. (2009). Animal Toxins: State of the Art - Perspectives in Health and Biotechnology. J. Venom. Anim. Toxins Incl. Trop. Dis. 15, 585–586. 10.1590/s1678-91992009000300021

[B85] Lu X. Williams J. A. Deadman J. J. Salmon G. P. Kakkar V. V. Wilkinson J. M. (1994). Preferential Antagonism of the Interactions of the Integrin αIIbβ3 with Immobilized Glycoprotein Ligands by Snake-Venom RGD (Arg-Gly-Asp) Proteins. Evidence Supporting a Functional Role for the Amino Acid Residues Flanking the Tripeptide RGD in Determining the Inhibitory Properties of Snake-Venom RGD Proteins. Biochem. J. 304, 929–936. 10.1042/bj3040929 7529494 PMC1137422

[B86] Machado L. E. S. F. De Paula V. S. Pustovalova Y. Bezsonova I. Valente A. P. Korzhnev D. M. (2018). Conformational Dynamics of a Cysteine-Stabilized Plant Defensin Reveals an Evolutionary Mechanism to Expose Hydrophobic Residues. Biochemistry 57, 5797–5806. 10.1021/acs.biochem.8b00753 30207151

[B87] Mackessy S. P. (2009). Handbook of Venoms and Toxins of Reptiles. Boca Raton: CRC Press. 10.1201/9781420008661

[B88] Marcinkiewicz C. Weinreb P. H. Calvete J. J. Kisiel D. G. Mousa S. A. Tuszynski G. P. (2003). Obtustatin: A Potent Selective Inhibitor of Alpha1beta1 Integrin *In Vitro* and Angiogenesis *In Vivo* . Cancer Res. 63, 2020–2023. 12727812

[B89] Marcinkiewicz C. Calvete J. J. Vijay-Kumar S. Marcinkiewicz M. M. Raida M. Schick P. (1999). Structural and Functional Characterization of EMF10, a Heterodimeric Disintegrin from Eristocophis Macmahoni Venom That Selectively Inhibits α5β1 Integrin. Biochemistry 38, 13302–13309. 10.1021/bi9906930 10529205

[B90] Marcinkiewicz C. Lobb R. R. Marcinkiewicz M. M. Daniel J. L. Smith J. B. Dangelmaier C. (2000). Isolation and Characterization of EMS16, a C-Lectin Type Protein from Echis Multisquamatus Venom, a Potent and Selective Inhibitor of the α2β1 Integrin. Biochemistry 39, 9859–9867. 10.1021/bi000428a 10933804

[B91] McLane M. A. Marcinkiewicz C. Vijay-Kumar S. Wierzbicka-Patynowski I. Niewiarowski S. (1998). Viper Venom Disintegrins and Related Molecules. Exp. Biol. Med. 219, 109–119. 10.3181/00379727-219-44322 9790167

[B92] McLane M. A. Vijay-Kumar S. Marcinkiewicz C. Calvete J. J. Niewiarowski S. (1996). Importance of the Structure of the RGD-Containing Loop in the Disintegrins Echistatin and Eristostatin for Recognition of αIIbβ3 and αvβ3 Integrins. FEBS Lett. 391, 139–143. 10.1016/0014-5793(96)00716-8 8706902

[B93] McLane M. Sanchez E. Wong A. Paquette-Straub C. Perez J. (2004). Disintegrins. Curr. Drug Targets Cardiovasc. Haematol. Disord. 4, 327–355. 10.2174/1568006043335880 15578957

[B94] Mcquade P. Knight L. C. Welch M. J. (2004). Evaluation of 64Cu- and 125I-Radiolabeled Bitistatin as Potential Agents for Targeting αvβ3 Integrins in Tumor Angiogenesis. Bioconjug. Chem. 15, 988–996. 10.1021/bc049961j 15366951

[B95] Moiseeva N. Bau R. Swenson S. D. Markland F. S. Choe J.-Y. Liu Z.-J. (2008). Structure of Acostatin, a Dimeric Disintegrin from Southern Copperhead (*Agkistrodon C* Contortrix), at 1.7 Å Resolution. Acta Crystallogr. D Biol. Cryst. 64, 466–470. 10.1107/S0907444908002370 18391413 PMC2631110

[B96] Momic T. Cohen G. Reich R. Arlinghaus F. T. Eble J. A. Marcinkiewicz C. (2012). Vixapatin (VP12), a C-Type Lectin-Protein from Vipera Xantina Palestinae Venom: Characterization as a Novel Anti-Angiogenic Compound. Toxins 4, 862–877. 10.3390/toxins4100862 23162702 PMC3496993

[B97] Moreno-Murciano M. P. (2003). Amino Acid Sequence and Homology Modeling of Obtustatin, a Novel Non-RGD-Containing Short Disintegrin Isolated from the Venom of Vipera Lebetina Obtusa. Protein Sci. 12, 366–371. 10.1110/ps.0230203 12538900 PMC2312415

[B98] Moura-da-Silva A. M. Furlan M. Caporrino M. Grego K. F. Portes-Junior J. Clissa P. B. (2011). Diversity of Metalloproteinases in Bothrops Neuwiedi Snake Venom Transcripts: Evidences for Recombination between Different Classes of SVMPs. BMC Genet. 12, 94. 10.1186/1471-2156-12-94 22044657 PMC3217872

[B99] Moura-da-Silva A. M. Theakston R. D. G. Crampton J. M. (1996). Evolution of Disintegrin Cysteine-Rich and Mammalian Matrix-Degrading Metalloproteinases: Gene Duplication and Divergence of a Common Ancestor rather Than Convergent Evolution. J. Mol. Evol. 43 (3), 263–269. 10.1007/bf02338834 8703092

[B100] Nagae M. Re S. Mihara E. Nogi T. Sugita Y. Takagi J. (2012). Crystal Structure of α5β1 Integrin Ectodomain: Atomic Details of the Fibronectin Receptor. J. Cel Biol. 197, 131–140. 10.1083/jcb.201111077 PMC3317794 22451694

[B101] Niewiarowski S. McLane M. A. Kloczewiak M. Stewart G. J. (1994). Disintegrins and Other Naturally Occurring Antagonists of Platelet Fibrinogen Receptors. Semin. Hematol. 31, 289–300. Available at: http://www.ncbi.nlm.nih.gov/pubmed/7831574 (Accessed June 30, 2021). 7831574

[B102] Nikai T. Taniguchi K. Komori Y. Masuda K. Fox J. W. Sugihara H. (2000). Primary Structure and Functional Characterization of Bilitoxin-1, a Novel Dimeric P-II Snake Venom Metalloproteinase from Agkistrodon Bilineatus Venom. Arch. Biochem. Biophys. 378, 6–15. 10.1006/abbi.2000.1795 10871038

[B103] Okuda D. Koike H. Morita T. (2002). A New Gene Structure of the Disintegrin Family: A Subunit of Dimeric Disintegrin Has a Short Coding Region. Biochemistry 41, 14248–14254. 10.1021/bi025876s 12450389

[B104] Okuda D. Nozaki C. Sekiya F. Morita T. (2001). Comparative Biochemistry of Disintegrins Isolated from Snake Venom: Consideration of the Taxonomy and Geographical Distribution of Snakes in the Genus Echis. J. Biochem. 129, 615–620. 10.1093/oxfordjournals.jbchem.a002898 11275562

[B105] Olfa K.-Z. José L. Salma D. Amine B. Najet S. A. Nicolas A. (2005). Lebestatin, a Disintegrin from Macrovipera Venom, Inhibits Integrin-Mediated Cell Adhesion, Migration and Angiogenesis. Lab. Invest. 85, 1507–1516. 10.1038/labinvest.3700350 16200076

[B106] Park D. Kang I. Kim H. Chung K. Kim D. S. Yun Y. (1998). Cloning and Characterization of Novel Disintegrins from Agkistrodon Halys Venom. Mol. Cell 8, 578–584. 9856345

[B107] Pfaff M. Mclane M. A. Beviglia L. Niewiarowski S. Timpl R. (1994). Comparison of Disintegrins with Limited Variation in the RGD Loop in Their Binding to Purified Integrins αIIbβ3, αVβ3 and α5β1 and in Cell Adhesion Inhibition. Cel Adhes. Commun. 2, 491–501. 10.3109/15419069409014213 7538018

[B108] Phillips D. Jennings L. Edwards H. (1980). Identification of Membrane Proteins Mediating the Interaction of Human Platelets. J. Cel Biol. 86, 77–86. 10.1083/jcb.86.1.77 PMC2110644 6893455

[B109] Pinheiro‐Aguiar R. do Amaral V. S. G. Pereira I. B. Kurtenbach E. Almeida F. C. L. (2020). Nuclear Magnetic Resonance Solution Structure of Pisum Sativum Defensin 2 Provides Evidence for the Presence of Hydrophobic Surface‐clusters. Proteins 88, 242–246. 10.1002/prot.25783 31294889

[B110] Rahman S. Aitken A. Flynn G. Formstone C. Savidge G. F. (1998). Modulation of RGD Sequence Motifs Regulates Disintegrin Recognition of αIIbβ3 and α5β1 Integrin Complexes. Biochem. J. 335, 247–257. 10.1042/bj3350247 9761721 PMC1219776

[B111] Sanz L. Bazaa A. Marrakchi N. Pérez A. Chenik M. Bel Lasfer Z. (2006). Molecular Cloning of Disintegrins from Cerastes vipera and *Macrovipera Lebetina* Transmediterranea Venom Gland cDNA Libraries: Insight into the Evolution of the Snake Venom Integrin-Inhibition System. Biochem. J. 395, 385–392. 10.1042/BJ20051678 16411889 PMC1422776

[B112] Sanz L. Chen R.-Q. Pérez A. Hilario R. Juárez P. Marcinkiewicz C. (2005). cDNA Cloning and Functional Expression of Jerdostatin, a Novel RTS-Disintegrin from Trimeresurus Jerdonii and a Specific Antagonist of the α1β1 Integrin. J. Biol. Chem. 280, 40714–40722. 10.1074/jbc.m509738200 16215260

[B113] Sanz-Soler R. Lorente C. Company B. Sanz L. Juárez P. Pérez A. (2012). Recombinant Expression of Mutants of the Frankenstein Disintegrin, RTS-Ocellatusin. Evidence for the Independent Origin of RGD and KTS/RTS Disintegrins. Toxicon 60, 665–675. 10.1016/j.toxicon.2012.05.010 22677804

[B114] Saviola A. J. Burns P. D. Mukherjee A. K. Mackessy S. P. (2016). The Disintegrin Tzabcanin Inhibits Adhesion and Migration in Melanoma and Lung Cancer Cells. Int. J. Biol. Macromolecules 88, 457–464. 10.1016/j.ijbiomac.2016.04.008 27060015

[B115] Scarborough R. M. Rose J. W. Hsu M. A. Phillips D. R. Fried V. A. Campbell A. M. (1991). Barbourin. A GPIIb-IIIa-specific Integrin Antagonist from the Venom of Sistrurus M. Barbouri. J. Biol. Chem. 266, 9359–9362. 10.1016/s0021-9258(18)92826-7 2033037

[B116] Scarborough R. M. Rose J. W. Naughton M. A. Phillips D. R. Nannizzi L. Arfsten A. (1993). Characterization of the Integrin Specificities of Disintegrins Isolated from American Pit viper Venoms. J. Biol. Chem. 268, 1058–1065. 10.1016/s0021-9258(18)54041-2 8419314

[B117] Schönthal A. H. Swenson S. D. Chen T. C. Markland F. S. (2020). Preclinical Studies of a Novel Snake Venom-Derived Recombinant Disintegrin with Antitumor Activity: A Review. Biochem. Pharmacol. 181, 114149. 10.1016/j.bcp.2020.114149 32663453

[B119] Senn H. Klaus W. (1993). The Nuclear Magnetic Resonance Solution Structure of Flavoridin, an Antagonist of the Platelet GP IIb-IIIa Receptor. J. Mol. Biol. 232, 907–925. 10.1006/jmbi.1993.1439 8355277

[B120] Shattil S. J. Newman P. J. (2004). Integrins: Dynamic Scaffolds for Adhesion and Signaling in Platelets. Blood 104, 1606–1615. 10.1182/blood-2004-04-1257 15205259

[B121] Sheu J. R. Lin C. H. Peng H. C. Huang T. F. (1996). Triflavin, an Arg-Gly-Asp-Containing Peptide, Inhibits the Adhesion of Tumor Cells to Matrix Proteins via Binding to Multiple Integrin Receptors Expressed on Human Hepatoma Cells. Exp. Biol. Med. 213, 71–79. 10.3181/00379727-213-44038 8820826

[B122] Shin J. Hong S.-Y. Chung K. Kang I. Jang Y. Kim D.-s. (2003). Solution Structure of a Novel Disintegrin, Salmosin, from Agkistrondon Halys Venom. Biochemistry 42, 14408–14415. 10.1021/bi0300276 14661951

[B123] Shiu J.-H. Chen C.-Y. Chen Y.-C. Chang Y.-T. Chang Y.-S. Huang C.-H. (2012). Effect of P to a Mutation of the N-Terminal Residue Adjacent to the Rgd Motif on Rhodostomin: Importance of Dynamics in Integrin Recognition. PLoS One 7, e28833. 10.1371/journal.pone.0028833 22238583 PMC3251565

[B124] Shiu J.-H. (2011). Structure, Dynamics, and Function Relationship of Rhodostomin Mutants and Variants:Insight into Their Interactions with Integrins. [Dissertation]. [Tainan (TW)]: National Cheng Kung University College of Medicine.

[B125] Siney E. J. Holden A. Casselden E. Bulstrode H. Thomas G. J. Willaime-Morawek S. (2017). Metalloproteinases ADAM10 and ADAM17 Mediate Migration and Differentiation in Glioblastoma Sphere-Forming Cells. Mol. Neurobiol. 54, 3893–3905. 10.1007/s12035-016-0053-6 27541285 PMC5443867

[B126] Smith K. J. Jaseja M. Lu X. Williams J. A. Hyde E. I. Trayer I. P. (1996). Three-Dimensional Structure of the RGD-Containing Snake Toxin Albolabrin in Solution, Based on 1H NMR Spectroscopy and Simulated Annealing Calculations. Int. J. Pept. Protein Res. 48, 220–228. 10.1111/j.1399-3011.1996.tb00835.x 8897089

[B127] Sohn Y.-D. Cho K.-S. Sun S.-A. Sung H.-J. Kwak K.-W. Hong S.-Y. (2008). Suppressive Effect and Mechanism of Saxatilin, a Disintegrin from Korean Snake (Gloydius Saxatilis), in Vascular Smooth Muscle Cells. Toxicon 52, 474–480. 10.1016/j.toxicon.2008.06.020 18625263

[B128] Stahle M. Jerome G. Nagaswami C. Weisel J. Hantgan R. (2002). Tirofiban Blocks Platelet Adhesion to Fibrin with Minimal Perturbation of GpIIb/IIIa Structure. Thromb. Haemost. 87, 910–917. 10.1055/s-0037-1613104 12038797

[B129] Staniszewska I. Walsh E. M. Rothman V. L. Gaathon A. Tuszynski G. P. Calvete J. J. (2009). Effect of VP12 and Viperistatin on Inhibition of Collagen Receptors: Dependent Melanoma Metastasis. Cancer Biol. Ther. 8, 1507–1516. 10.4161/cbt.8.15.8999 19502781

[B130] Surażyński A. Sienkiewicz P. Wołczyński S. Pałka J. (2005). Differential Effects of Echistatin and Thrombin on Collagen Production and Prolidase Activity in Human Dermal Fibroblasts and Their Possible Implication in β1-integrin-mediated Signaling. Pharmacol. Res. 51, 217–221. 10.1016/j.phrs.2004.08.004 15661571

[B132] Szabo A. M. Howell N. R. Pellegrini P. Greguric I. Katsifis A. (2012). Development and Validation of Competition Binding Assays for Affinity to the Extracellular Matrix Receptors, αvβ3 and αIIbβ3 Integrin. Anal. Biochem. 423, 70–77. 10.1016/j.ab.2011.12.046 22285979

[B133] Takagi J. Petre B. M. Walz T. Springer T. A. (2002). Global Conformational Rearrangements in Integrin Extracellular Domains in Outside-In and Inside-Out Signaling. Cell 110, 599–611. 10.1016/S0092-8674(02)00935-2 12230977

[B134] Takeda S. Takeya H. Iwanaga S. (2012). Snake Venom Metalloproteinases: Structure, Function and Relevance to the Mammalian ADAM/ADAMTS Family Proteins. Biochim. Biophys. Acta (Bba) - Proteins Proteomics 1824, 164–176. 10.1016/j.bbapap.2011.04.009 21530690

[B135] Takeya H. Nishida S. Miyata T. Kawada S. Saisaka Y. Morita T. (1992). Coagulation Factor X Activating Enzyme from Russell's viper Venom (RVV-X). A Novel Metalloproteinase with Disintegrin (Platelet Aggregation Inhibitor)-like and C-Type Lectin-Like Domains. J. Biol. Chem. 267, 14109–14117. 10.1016/s0021-9258(19)49685-3 1629211

[B136] Tcheng J. E. O’Shea J. C. (2002). Eptifibatide: A Potent Inhibitor of the Platelet Receptor Integrin Glycoprotein IIb/IIIa. Expert Opin. Pharmacother. 3, 1199–1210. 10.1517/14656566.3.8.1199 12150697

[B138] Trikha M. Rote W. E. Manley P. J. Lucchesi B. R. Markland F. S. (1994). Purification and Characterization of Platelet Aggregation Inhibitors from Snake Venoms. Thromb. Res. 73, 39–52. 10.1016/0049-3848(94)90052-3 8178312

[B139] Trim C. M. Byrne L. J. Trim S. A. (2021). Utilisation of Compounds from Venoms in Drug Discovery. 1st ed. Amsterdam: Elsevier B.V, 1–66. 10.1016/bs.pmch.2021.01.001 34147202

[B140] Tselepis V. H. Green L. J. Humphries M. J. (1997). An RGD to LDV Motif Conversion within the Disintegrin Kistrin Generates an Integrin Antagonist That Retains Potency but Exhibits Altered Receptor Specificity. J. Biol. Chem. 272, 21341–21348. 10.1074/jbc.272.34.21341 9261147

[B141] Tur-Fu H. Chao-Zong L. Chaoho O. Che-Ming T. (1991). Halysin, an Antiplatelet Arg-Gly-Asp-Containing Snake Venom Peptide, as Fibrinogen Receptor Antagonist. Biochem. Pharmacol. 42, 1209–1219. 10.1016/0006-2952(91)90256-5 1888330

[B142] Uzair B. Atlas N. Malik S. B. Jamil N. Ojuolape S. T. Rehman M. U. (2018). Snake Venom as an Effective Tool Against Colorectal Cancer. Ppl 25, 626–632. 10.2174/0929866525666180614112935 29921196

[B143] Van Agthoven J. F. Xiong J.-P. Alonso J. L. Rui X. Adair B. D. Goodman S. L. (2014). Structural Basis for Pure Antagonism of Integrin αVβ3 by a High-Affinity Form of Fibronectin. Nat. Struct. Mol. Biol. 21, 383–388. 10.1038/nsmb.2797 24658351 PMC4012256

[B145] Walsh E. M. Marcinkiewicz C. (2011). Non-RGD-Containing Snake Venom Disintegrins, Functional and Structural Relations. Toxicon 58, 355–362. 10.1016/j.toxicon.2011.07.004 21801741

[B146] Wermelinger L. S. Geraldo R. B. Frattani F. S. Rodrigues C. R. Juliano M. A. Castro H. C. (2009). Integrin Inhibitors from Snake Venom: Exploring the Relationship between the Structure and Activity of RGD-Peptides. Arch. Biochem. Biophys. 482, 25–32. 10.1016/j.abb.2008.11.023 19101499

[B147] Wu P.-L. Lin C.-C. Lin T.-H. Lee M.-S. Wu W.-G. (2016). Distal M Domain of Cobra ADAM-like Metalloproteinase Mediates the Binding of Positively Charged Cysteine-Rich Domain to αvβ3 Integrin in the Suppression of Cell Migration. Toxicon 118, 1–12. 10.1016/j.toxicon.2016.04.034 27090013

[B148] Wu W. B. Chang S. C. Liau M.-Y. Huang T.-F. (2001). Purification, Molecular Cloning and Mechanism of Action of Graminelysin I, a Snake-Venom-Derived Metalloproteinase That Induces Apoptosis of Human Endothelial Cells. Biochem. J. 357, 719–728. 10.1042/bj3570719 11463342 PMC1222001

[B149] Xia X. Ma Y. Xue S. Wang A. Tao J. Zhao Y. (2013). Cloning and Molecular Characterization of BumaMPs1, a Novel Metalloproteinases from the Venom of Scorpion Buthus Martensi Karsch. Toxicon 76, 234–238. 10.1016/j.toxicon.2013.10.006 24125658

[B150] Xiao T. Takagi J. Coller B. S. Wang J.-H. Springer T. A. (2004). Structural Basis for Allostery in Integrins and Binding to Fibrinogen-Mimetic Therapeutics. Nature 432, 59–67. 10.1038/nature02976.Structural 15378069 PMC4372090

[B151] Xiong J.-P. Stehle T. Zhang R. Joachimiak A. Frech M. Goodman S. L. (2002). Crystal Structure of the Extracellular Segment of Integrin αVβ3 in Complex with an Arg-Gly-Asp Ligand. Science 296 (5565), 151–155. 10.1126/science.1069040 11884718

[B152] Xiong J. P. Stehle T. Diefenbach B. Zhang R. Dunker R. Scott D. L. (2001). Crystal Structure of the Extracellular Segment of Integrin Alpha Vbeta3. Science 294, 339–345. 10.1126/science.1064535 11546839 PMC2885948

[B153] Yang R.-S. Tang C.-H. Chuang W.-J. Huang T.-H. Peng H.-C. Huang T.-F. (2005). Inhibition of Tumor Formation by Snake Venom Disintegrin. Toxicon 45, 661–669. 10.1016/j.toxicon.2005.01.013 15777962

[B154] Yeh C.-H. Peng H.-C. Yang R.-S. Huang T.-F. (2001). Rhodostomin, A Snake Venom Disintegrin, Inhibits Angiogenesis Elicited by Basic Fibroblast Growth Factor and Suppresses Tumor Growth by A Selective αvβ3Blockade of Endothelial Cells. Mol. Pharmacol. 59, 1333–1342. 10.1124/mol.59.5.1333 11306719

